# Assessment and molecular characterization of *Bacillus cereus* isolated from edible fungi in China

**DOI:** 10.1186/s12866-020-01996-0

**Published:** 2020-10-14

**Authors:** Chengcheng Liu, Pengfei Yu, Shubo Yu, Juan Wang, Hui Guo, Ying Zhang, Junhui Zhang, Xiyu Liao, Chun Li, Shi Wu, Qihui Gu, Haiyan Zeng, Youxiong Zhang, Xianhu Wei, Jumei Zhang, Qingping Wu, Yu Ding

**Affiliations:** 1 Guangdong Institute of Microbiology, Guangdong Academy of Science, State Key Laboratory of Applied Microbiology Southern China, Guangdong Provincial Key Laboratory of Microbial Safety and Health, Guangdong Open Laboratory of Applied Microbiology, Xianlie Zhong Road 100#, 58th Building, Guangzhou, 510070 China; 2 Department of Food Science and Technology, Institute of Food Safety and Nutrition, Jinan University, Huangpu Ave. 601, Guangzhou, 510632 China; 3 College of Food Science, South China Agricultural University, Guangzhou, China

**Keywords:** *Bacillus cereus*, Edible fungi, Prevalence, Antibiotic resistance, Genetic diversity

## Abstract

**Background:**

*Bacillus cereus* is a foodborne pathogen commonly found in nature and food and can cause food spoilage and health issues. Although the prevalence of *B. cereus* in foods has been reported worldwide, the extent of contamination in edible fungi, which has become increasingly popular as traditional or functional food, is largely unknown. Here we investigated the prevalence, toxin genes’ distribution, antibiotic resistance, and genetic diversity of *B. cereus* isolated from edible fungi in China.

**Results:**

Six hundred and ninety-nine edible fungi samples were collected across China, with 198 (28.3%) samples found to be contaminated by *B. cereus*, with an average contamination level of 55.4 most probable number (MPN)/g. Two hundred and forty-seven *B. cereus* strains were isolated from the contaminated samples. Seven enterotoxin genes and one cereulide synthetase gene were detected. The detection frequencies of all enterotoxin genes were ≥ 80%, whereas the positive rate of the *cesB* gene in *B. cereus* was 3%. Most isolates were resistant to penicillins, β-lactam/β-lactamase inhibitor combinations, cephems, and ansamycins, but were susceptible to penems, aminoglycosides, macrolides, ketolide, glycopeptides, quinolones, phenylpropanol, tetracyclines, lincosamides, streptogramins, and nitrofurans. Meanwhile, 99.6% of all isolates displayed multiple antimicrobial resistance to three or more classes of antimicrobials. Using genetic diversity analysis, all isolates were defined in 171 sequence types (STs), of which 83 isolates were assigned to 78 new STs.

**Conclusions:**

This study provides large-scale insight into the prevalence and potential risk of *B. cereus* in edible fungi in China. Approximately one-third of the samples were contaminated with *B. cereus*, and almost all isolates showed multiple antimicrobial resistance. Detection frequencies of all seven enterotoxin genes were equal to or more than 80%. These new findings may indicate a need for proper pre-/post-processing of edible fungi to eliminate *B. cereus*, thereby preventing the potential risk to public health.

## Background

Foodborne disease is an important public health issue worldwide, which is among the leading causes of morbidity and mortality [1–4]. Data from the foodborne disease outbreak surveillance and reporting system showed 5021 foodborne disease outbreaks were reported between 2001 to 2010 in China [5]. These outbreaks involved 140,101 cases and 1427 deaths, with fatality rate of 1.0%, and pathogenic microorganisms accounted for most of the incidents and cases [5]. Ninety-eight foodborne disease outbreaks were reported in Shandong, China in 2014, involving 1238 patients and four deaths, mostly caused by pathogenic microorganisms [6]. The top five bacterial pathogens responsible for foodborne outbreaks from 2011 to 2016 in China were *Vibrio parahaemolyticus*, *Salmonella spp.*, *Staphylococcus aureus* (enterotoxin), *Bacillus cereus*, and diarrheagenic *Escherichia coli* [7]. Notably, there had been 4342 foodborne cases caused by *B. cereus* during these 6 years.

*B. cereus* is a Gram-positive, spore-forming bacterium widespread in natural environment [8, 9]. It can cause two types of food poisoning in humans, including diarrheal and emetic syndromes, along with a variety of local and systemic infections, such as endophthalmitis, endocarditis, meningitis, osteomyelitis, wound infections, and septicemia [10, 11]. It causes diarrhea and emesis via the production of diarrheal enterotoxins and emetic toxin, respectively. The enterotoxins include hemolysin BL (Hbl), nonhemolytic enterotoxin (Nhe), and the single-protein enterotoxin, cytotoxin K (CytK) [9, 12, 13]. Hbl and Nhe are tripartite toxins, in which all three components are necessary for maximal cytotoxic activity [14, 15]. The emetic toxin cereulide is a cyclic depsipeptide toxin encoded by the *ces* gene cluster [16, 17]. Emetic symptoms usually occur within 0.5-6 h after consuming contaminated foods [9]. Cereulide is pre-formed in food and is difficult to inactivate either during food processing or in the gastrointestinal tract, owing to its high resistance to heat treatments, extreme pH conditions, and enzymolysis [9, 16].

To date, antibiotic treatment is one choice in clinical treatment for severe food poisoning and other body or tissue infections caused by *B. cereus* [18–20]. However, studies have reported *B. cereus* to be resistant to different antibiotic agents, thus constituting a fundamental problem worldwide [21–23]. Information is therefore necessary for an antibiotic resistance profile of this bacterium.

*B. cereus* has been isolated from many foodstuffs, such as rice, pasta, vegetables, meat and meat products, soup, and milk and other dairy products [24–29]. However, the actual incidence rate of *B. cereus* in edible fungi remains largely unknown. From culinary perspective, edible fungi are consumed worldwide. The nutritional properties and health benefits of ‘medicinal’ edible fungi have been known in China for over 2000 years. Besides, edible fungi have been a part of traditional Chinese medicine, and have also been used in Japan and Malaysia [30]. *B. cereus* contamination could lead to spoilage of edible fungi and cause food poisoning. It is therefore necessary to investigate the prevalence of *B. cereus* in edible fungi.

The objective of this study was to investigate the contamination level and toxin gene distribution, including seven diarrheal enterotoxin genes and one emetic toxin gene across the 247 *B. cereus* strains isolated from edible fungi in China. In addition, we characterized the isolates based on their antimicrobial resistance and genotypic diversity. Thereby, we provided a systematic risk assessment for *B. cereus* isolated from edible fungi in China.

## Results

### Contamination of edible fungi with *B. cereus*

The prevalence of *B. cereus* in 699 edible fungi samples examined in this study is shown in Table 1. *B. cereus* was detected in 28.3% (198/699) of all samples collected and 247 isolates were isolated from these contaminated samples. Average contamination level for the positive samples was 55.4 MPN/g. 20.6% (45/218) of *Flammulina velutipes*, 34.5% (39/113) of *Pleurotus ostreatus*, 57.8% (63/109) of *Lentinus edodes*, 14.7% (14/95) of *Pleurotus eryngii*, 16.0% (15/94) of *Hypsizygus marmoreus,* and 31.4% (22/70) of other species were contaminated with *B. cereus*, respectively. The contamination levels of 81.8% (162/198) of positive samples ranged from 3 to 1100 MPN/g, with that in five samples (5/198, 2.5%) exceeding 1100 MPN/g.

**Table 1:** Prevalence and contamination level of *B. cereus* in different edible fungi

Sample	Contamination rate (%) ^**a**^	MPN value (MPN/g) ^**b**^			Positive sample contamination level (MPN/g) ^**c**^
		MPN ***< 3***	3 ≤ MPN ***<*** 1100	1100 ≤ MPN	
*Flammulina velutiper*	20.6 (45/218)	13/45 (28.9**%**)	31/45 (68.9**%**)	1/45 (2.2**%**)	55.5
*Pleurotus ostreatus*	34.5 (39/113)	4/39 (10.3**%**)	33/39 (84.6**%**)	2/39 (5.1**%**)	91.7
*Lentinus edodes*	57.8 (63/109)	5/63 (7.9**%**)	58/63 (92.1**%**)	0/63 (0.0**%**)	24.6
*Pleurotus eryngii*	14.7 (14/95)	4/14 (28.6**%**)	10/14 (71.4**%**)	0/14 (0.0**%**)	6.8
*Hypsizygus marmoreus*	16.0 (15/94)	2/15 (13.3**%**)	13/15 (86.7**%**)	0/15 (0.0**%**)	49.9
Other species	31.4 (22/70)	3/22 (13.6**%**)	17/22 (77.3**%**)	2/22 (9.1**%**)	113.3
Total	28.3 (198/699)	31/198 (15.7**%**)	162/198 (81.8**%**)	5/198 (2.5**%**)	55.4

^a^Contamination rate = Number of positive samples/Total samples;

^b^MPN value (MPN/g) = Most probable number of *B. cereus* per gram sample;

^c^Positive sample contamination levels (MPN/g) = Sum of MPN values for positive sample/weight of positive samples.

### Distribution of toxin genes

**Fig. 1:**
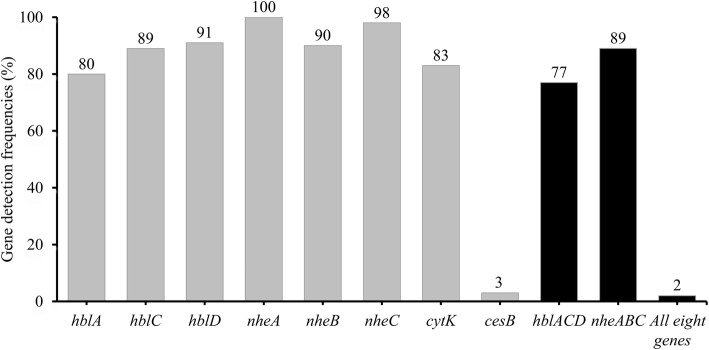
Detection frequencies of toxin genes in *B. cereus* from edible fungi. The number at the top of the bars represents positive rate of corresponding toxin genes. *hblACD* and *nheABC* show the strains to be simultaneously positive for *hblA*, *hblC*, and *hblD*; or *nheA*, *nheB*, and *nheC*, respectively. “All eight genes” represents the strains with all the detected toxin genes

The positive rate of all seven enterotoxin genes was ≥80%. Specifically, 80, 89, 91, 100, 90, 98, and 83% of any of the isolates harbored *hblA*, *hblC*, *hblD*, *nheA*, *nheB*, *nheC*, and *cytK*, respectively (Fig. 1; Additional file 1: Table S1). In contrast, 3% of the isolates were positive for the cereulide synthetase gene *B* (*cesB*). All isolates were positive for at least two toxin genes, and 157 (64%) isolates harbored seven or more toxin genes. Among the 30 different gene profiles, the most commonly observed gene profile was *hblA-hblC-hblD-nheA-nheB-nheC-cytK*, possessed by 62% of the isolates.

### Antibiotic susceptibility of *B. cereus* in edible fungi

**Fig. 2:**
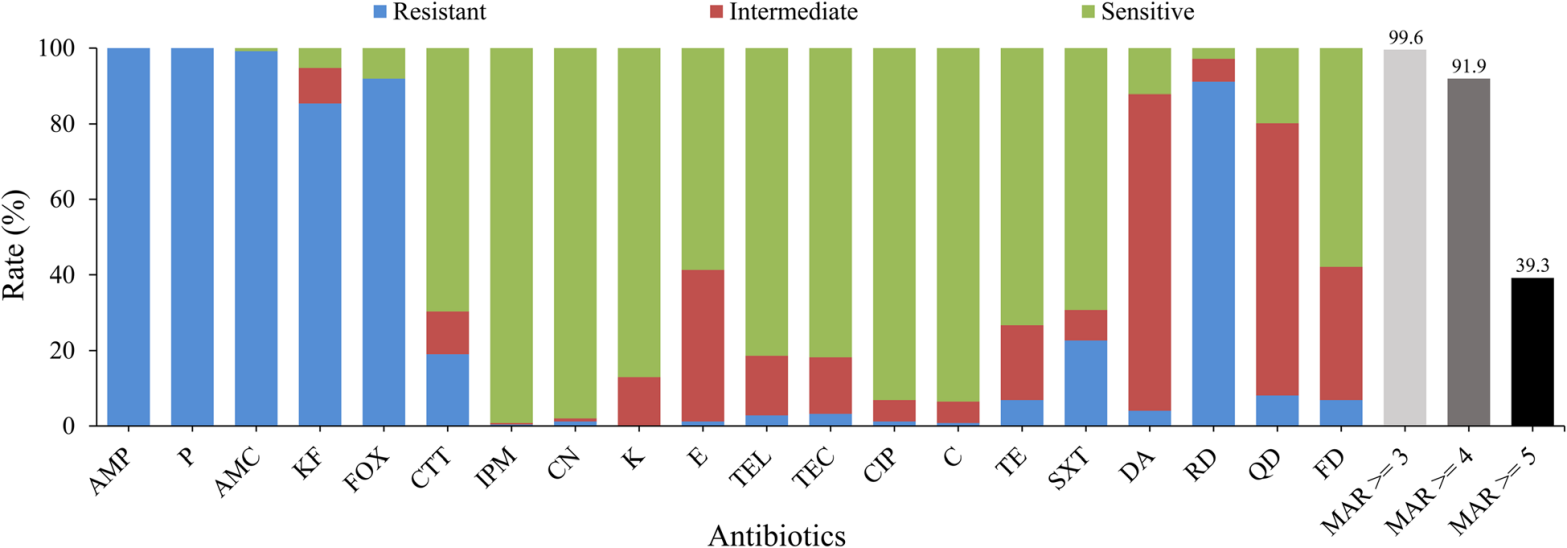
Antimicrobial resistance of *B. cereus* from edible fungi. The blue, red, and green bars represent the proportion of resistance, intermediate resistance, and sensitive strains, respectively. The light gray, gray and black bars represents the proportion of strains with multiple antimicrobial resistance (MAR) to at least three, four, and five classes, respectively

The antibiotic susceptibility results of 247 *B. cereus* isolates to 20 selected antimicrobials are shown in Fig. 2. The diameters of inhibition zones in the antimicrobial susceptibility test are included in Additional file 2: Table S2. All isolates were resistant to ampicillin (AMP) and penicillin (P). Most isolates were resistant to amoxicillin-clavulanic acid (AMC; 99.2%), cephalothin (KF; 85.4%), cefoxitin (FOX; 91.9%), and rifampin (RD; 91.1%). Over 93% of the isolates showed susceptibility to imipenem (IPM; 99.2%), gentamicin (CN; 98.0%), ciprofloxacin (CIP; 93.1%), and chloramphenicol (C; 93.5%). Moreover, most isolates showed intermediate resistance to clindamycin (DA; 83.8%) and quinupristin-dalfopristin (QD; 72.1%) (Additional file 3: Table S3).

All isolates were resistant to more than two antibiotics. Five isolates (2.0%) were resistant to ≥10 antibiotics, and 33 isolates (13.4%) showed resistance to at least eight antibiotics (Additional file 4: Table S4). For the most common antimicrobial resistant pattern, 33.6% of the isolates harbored the profile AMP-P-AMC-KF-FOX-RD (Additional file 4: Table S4). In addition, 99.6% of all isolates displayed multiple antimicrobial resistance to three and more classes of antimicrobials (Fig. 2). Approximately 91.9 and 39.3% of the isolates showed multiple antimicrobial resistance to more than three or four classes of antimicrobials, respectively (Fig. 2).

### Multi-locus sequence typing and clustering of the isolates

**Fig. 3:**
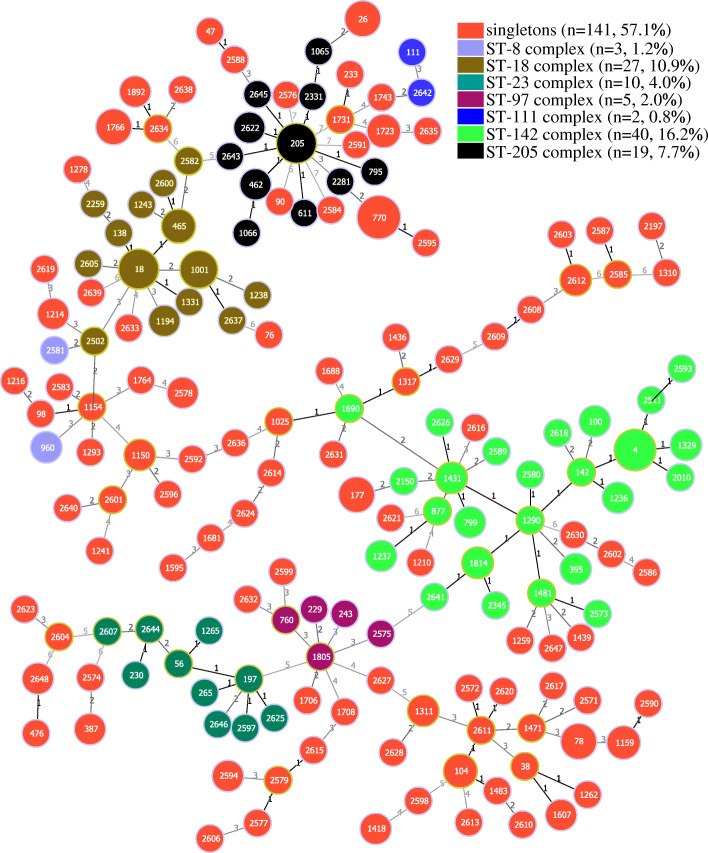
Minimum spanning tree and genetic diversity of *B. cereus* from edible fungi. Colors inside the circles represent clonal complexes and singletons. The numbers inside the circles represent different sequence types (STs). Gradation of the line color and corresponding number along the line represent the variation of seven loci between the two strains at both ends of the line. Dominant STs are represented by circles with larger diameters

Of the 171 STs assigned to all isolates, ST770 was the most prevalent but only included 11 isolates, and 135 STs (78.9%) included only one isolate. Eighty-three (33.6%) of the 247 isolates belonged to one of 78 novel STs. Of all the isolates, 141 singletons and seven clonal complexes (CCs) were assigned. The most prevalent CC was the ST-142 complex, which included 40 isolates (Additional file 4: Table S4). The ST-8, ST-18, ST-23, ST-97, ST-111, and ST-205 complexes included 3, 27, 10, 5, 2, and 19 isolates, respectively (Fig. 3; Additional file 4: Table S4). Regarding the phylogenetic relationship, all isolates were grouped into ten clusters with the cut-off value of 43% similarity (Additional file 5: Figure S1) and the cluster numbers were defined depended on the description of Guinebretière et al. [31]. Cluster III contained the most strains. Cluster IX only contained one isolate identified in this study, and cluster X only possessed *Bacillus anthracis* ATCC4728, indicating these strains were far away from the others. Isolates in cluster I were phylogenetically map together with *Bacillus pseudomycoides* DSM 12442, and isolates in cluster III were map together with *Bacillus weihenstephanensis* WSBC 10204 and *Bacillus cytotoxicus* NVH 391-98, which indicating that these strains may be phylogenetically close with each other.

## Discussion

*B. cereus* is an invasive and opportunistic pathogen that can cause gastrointestinal diseases and severe nosocomial infections. To date, prevalence studies of pathogenic *B. cereus* isolated from edible fungi are limited. We therefore investigated the contamination characteristics of *B. cereus* from edible fungi collected between 2011 to 2016. *B. cereus* was present in 28.3% of edible fungi samples collected from major cities of China. This suggested that more attention should be paid to the extent of contamination in edible fungi. Contamination may occur at any point during cultivation, processing, storage, and sale of the edible fungi. Compared to previous surveys with other foodstuffs (8.2% in infant formula, 3.8% in rice flour, and 9% in ready-to-eat foods), the contamination rate of *B. cereus* isolated from edible fungi was higher [32, 33]. Unlike our previous studies which showed another important foodborne pathogen, *Listeria monocytogenes*, to be highly associated with *F. velutipes* [34, 35]; here, *B. cereus* was frequently detected in *L. edodes.* Since > 50% of the *L. edodes* samples were positive for *B. cereus*, further studies regarding the contamination source would be recommended, considering the *B. cereus* strains to possibly be the contamination sources for the environment and other foodstuffs. In addition, the close correlation between *B. cereus* and *L. edodes* should also be investigated. Although there is no specified standard for the microbiological limits of *B. cereus* in edible fungi in China, the amounts between 10^3^ and 10^5^ CFU/g of *B. cereus* in ready-to-eat foods are “acceptable”, but not “satisfactory”, according to the Microbiological Guidelines for Food of Hong Kong, China [36], hence suggesting a potential for public health risk. Studies have shown that more than 100 CFU/g of *B. cereus* in food samples may lead to foodborne infection [37, 38]. In this study, contamination levels in 2.5% of positive samples exceeded 1100 MPN/g (Table 1). A *B. cereus* strain (strain number 49-1), isolated from one of these samples, harbored the *cesB* gene and was simultaneously identified as a ST26 strain. The reported emetic type strains F4810/72 and NC7401 [39, 40], were also identified as ST26 based on their complete genome sequences in the NCBI genome database. These results indicated a potential hazard of these contaminated edible fungi and the need for adequate cleaning and heating measures before consumption.

According to toxin gene distribution, 30 different toxin gene profiles were identified from the isolates. The detection frequency of *nheABC* (89%) was similar to the findings in previous studies reporting that over 90% of isolates, from a variety of food samples, contained the *nheABC* gene cluster [28, 29, 41]. Besides, the detection frequencies of *nheC*, *nheB*, and *nheA* were close to that of *B. cereus* isolated from different food samples (100, 99, and 96%, respectively) [38], in line with these three genes being generally prevalent in *B. cereus* isolates. The detection frequencies of *nheA*, *nheB*, and *nheC* were not equal, which is also consistent with the previous studies [38, 42, 43]. These three genes were not always simultaneously found in the same isolate for unknown reasons, as were the genes *hblA*, *hblC*, and *hblD*. One possible explanation could be that the general primers used for detection cannot cover the polymorphism of target genes in different strains and affected the amplification efficiency in PCR. One hundred and ninety-one (77%) isolates simultaneously carried the three hemolysin genes, *hblA, hblC,* and *hblD*, which had higher detection frequencies than in other foodstuffs (55% in raw milk samples, and 45% in pasteurized milk samples) [27, 28]. The most common toxin gene profile among all isolates (62%) was *hblA-hblC-hblD-nheA-nheB-nheC-cytK*, revealing that *B. cereus* isolated from edible fungi mainly harbored the diarrheal enterotoxin-encoding genes, in line with previous research [44]. Although the detection frequency of *cesB* is lower than that of all the enterotoxin genes tested, it is still higher than that in a previous study [32], indicating the potential virulence of these isolates.

*B. cereus* has been reported to be resistant to different antimicrobials [45–47]. In this study, we found that *B. cereus* isolates from edible fungi were mainly resistant to β-lactams (except cefotetan and imipenem) and ansamycins, especially ampicillin and penicillin. Previous studies had shown that *B. cereus* harbors the antibiotic resistance genes encoding β-lactamases [11, 23, 48, 49]. Therefore, *B. cereus* has the ability to degrade β-lactams and show resistant characteristics [23, 28, 29, 48, 49]. In contrast, *B. cereus* isolated from edible fungi were still sensitive to aminoglycosides, ketolide, glycopeptides, quinolones, and phenylpropanol antibiotics, in agreement with some previous studies [24, 27–29, 50]. Ciprofloxacin, clindamycin, and aminoglycoside antibiotics have been highly effective in the treatment of diseases caused by *B. cereus* [11, 51–53]. However, 83.8% of the isolates in this study showed intermediate resistance to clindamycin, indicating that clindamycin may be less effective at inhibiting these isolates. Lee et al. have reported that *B. cereus* isolates from Sunsik were resistant to ampicillin, cefoxitin, and penicillin, which were in concordance with our results. However, their isolates were fully sensitive to chloramphenicol, gentamicin, imipenem, sulfamethoxazole, and tetracycline [54], whereas 22.7% of our isolates showed resistance to sulfamethoxazole. Luna et al. [55] and Ikram et al. [56] had reported *B. cereus* to be resistant to ampicillin, penicillin, and sensitive to chloramphenicol, ciprofloxacin, and gentamicin, similar to our findings; however, 72.1% of our isolates showed intermediate resistance to quinupristin-dalfopristin and 91.1% of our isolates showed resistance to rifampin. These differences may be caused by the differences in sample sources, and sampling sites. Remarkably, we found 99.6% of all the isolates to be simultaneously resistant to antibiotics from three or more antimicrobial classes, with 91.9 and 39.3% of the isolates showing multiple antimicrobial resistance to more than three or four classes, respectively. The emergence of multiple antimicrobial resistant bacteria has led to a reduction in the types and quantities of effective antibacterial drugs available for pathogen elimination, and may further lead to a decline in the effectiveness of clinical treatment for bacterial infection. The potential hazards of multiple antimicrobial resistant isolates may need further investigation.

Of the isolates studied, 33.6% (83/247) were assigned to new STs, indicating the diverse bacterial resources not yet identified in this study. Of the 171 STs, 135 had only one isolate; thus, at least 54.7% of the isolates had a unique ST from each other, revealing the genetic diversity of isolates from edible fungi. The most prevalent ST was ST770, and the dominant CC was the ST-142 clonal complex, contradicting with previous studies [57, 58]. The reason for this phenomenon may be the difference in isolation sources of the strains. From clustering results in evolutionary analysis (Additional file 5: Figure S1), we could find specific characteristics of antibiotic resistance of the isolates. Clusters IX and X possessed only one strain each. Only isolates in cluster VI showed no QD (quinupristin-dalfopristin) resistance. Isolates in cluster I, III, IV and V showed resistance to TEL (telithromycin), and isolates in cluster III, V and VI showed resistance to TEC (teicoplanin). There were only 20, 7, and 8 isolates showing resistance to QD, TEL, and TEC. Three (isolates 49-1, 633-1C, and 1284) of the seven *cesB*-positive isolates identified in this study were grouped in cluster III, and they were likely to be gathered with the *B. cereus* emetic type strains NC7401 and F4810/72. Thus, the pathogenic potential of these strains may be higher than the others, and need to be investigated in future. Interestingly, the other four *cesB*-positive isolates were randomly distributed in different branches, suggesting the phylogenetic relationship between them to be distant, probably evolved from different origins.

## Conclusion

The prevalence of *B. cereus* in about one third of the edible fungi samples in China indicated a source of potential infectious exposure to the consumers. Edible fungi samples with a *B. cereus* contamination level of ≥1100 MPN/g suggest the importance of performing extensive cleaning and/or processing before consumption. Of the 247 isolates, 171 STs were assigned, of which 135 STs only contained one isolate and 78 new STs were defined. Moreover, 64% of the isolates harbored all the detected (seven) enterotoxins, among which four isolates further harbored the cereulide synthetase gene *cesB*. Three *cesB*-positive strains were identified as the same ST as *B. cereus* clinical emetic type strains and simultaneously phylogenetically clustered together with them. Isolates exhibiting multiple antimicrobial resistance and multiple toxin genes may indicate potential health risks related to *B. cereus* from edible fungi. Therefore, further studies would be recommended to identify the pathogenic potential of these strains.

## Methods

### Edible fungi sample collection

From July 2011 to January 2016, 699 edible fungi samples (including 218 *Flammulina velutipes*, 113 *Pleurotus ostreatus*, 109 *Lentinus edodes*, 95 *Pleurotus eryngii*, 94 *Hypsizygus marmoreus*, and 70 other species) were obtained from 39 cities, covering all the provincial capital cities of China. The details of sampling positions are shown in Additional file 6: Figure S2.

The samples were stored in sealed bags and placed in cooling boxes (1-4 °C). They were transported to the laboratory within 12 h and analyzed immediately according to the general sample collection guidelines of the National Food Safety Standard [59].

### Identification of *B. cereus* in edible fungi

All samples were subjected to qualitative and quantitative analysis for *B. cereus,* according to the *B. cereus* test standards given by the National Food Safety Standard [60, 61] with some modifications, as previously described [28, 29]. Twenty-five grams of the collected samples were cut and mixed with 225 mL phosphate buffered saline (PBS, 0.01 mol/L) under aseptic conditions. Then, 10- and 100-fold dilutions of the homogenized solution were prepared. One milliliter of each dilution was inoculated into nine milliliters of tryptic soy broth (TSB) with polymyxin B (Huankai, Guangzhou, China) in different tubes. Each experiment was repeated in triplicate in parallel. The tubes were incubated at 30 °C for 8-18 h. The samples were streaked onto mannitol egg yolk polymyxin (MYP) agar plates (Huankai, Guangzhou, China) and incubated at 30 °C for 24-48 h.

The presumptive colonies, in pink color with a pink halo, were selected and streaked on the chromogenic *B. cereus* agar plates (Huankai, Guangzhou, China) and then incubated at 30 °C for 24 h. The blue-green colonies were selected and streaked on the nutrient agar plates (Huankai, Guangzhou, China). The selected colonies were incubated and used for further analysis, including biochemical identification, parasporal crystal observation, hemolysis test, MYP agar plate test, root growth observation, catalase test, motility test, nitrate reduction test, casein decomposition test, lysozyme tolerance test, glucose utilization test, and acetyl methyl alcohol test, described in the National Food Safety Standard [60, 61] and the Bacteriological Nnalytical Manuals of the U.S. Food and Drug Administration [62]. The isolate, which 1) produces large Gram-positive rods with spores that does not swell the sporangium; 2) produces lecithinase and does not ferment mannitol on MYP agar; 3) grows and produces acid from glucose anaerobically; 4) reduces nitrate to nitrite (a few strains may be negative); 5) produces acetylmethylcarbinol (VP-positive); 6) decomposes L-tyrosine; 7) grows in the presence of 0.001% lysozyme; 8) is actively motile and strongly hemolytic; 9) does not produce rhizoid colonies or protein toxin crystals, can be identified as *B. cereus*. The most probable number (MPN), which represents the number of corresponding bacteria most likely to be present per gram of sample, of *B. cereus* for the samples was determined according to the National Food Safety Standard [60, 61], the Bacteriological Nnalytical Manuals of the U.S. Food and Drug Administration [62], and previous studies [29, 63, 64].

### Detection of toxin genes

Genomic DNA was extracted from *B. cereus* using a HiPure DNA Extraction Kit (Magen, Guangzhou, China), according to the manufacturer’s instructions. Eight toxin genes, including seven enterotoxin genes (*nheA*, *nheB*, *nheC, hblC*, *hblD*, *hblA,* and *cytK*) and a cereulide synthetase gene *B* (*cesB*) were detected. The primers used for the detection of toxin genes and their annealing temperature are shown in Additional file 7: Table S5.

The polymerase chain reaction (PCR) mixture (25 μL) comprised of 50 ng genomic DNA, 0.5 μL of each primer solution (concentration 10 μM), and 12.5 μL PCR Premix Taq™ (Takara, China). The amplification process included: initial denaturation at 94 °C for 5 min, 30 cycles of 94 °C for 1 min, 55 °C or 58 °C (58 °C for *cesB* and 55 °C for the other toxin genes) for 1 min, 72 °C for 1 min, and a final 10 min extension at 72 °C [17, 43, 65, 66]. PCR was conducted in a TONE-96G PCR Thermal Cycler (Analytik Jena, Jena, Germany). Amplicons were subjected to electrophoresis using 1.5% agarose gel containing 0.01% Gold View. The gels were visualized by a UV Imaging System (Bio-Rad, Hercules, USA). A 2000-bp DNA ladder (Dongsheng, Guangzhou, China) was used as a molecular weight marker. The images were captured in TIFF file format for further analysis.

### Antimicrobial susceptibility test

Antimicrobial susceptibility was evaluated using the Kirby-Bauer disk diffusion method on Mueller-Hinton (MH) agar for 20 selected antimicrobial drugs belonging to 16 different classes [28, 29, 67]. The details of antimicrobial agents (Oxoid, Basingstoke, UK) are shown in Fig. 2, and Additional file 3: Table S3. Following the methods of CLSI [67] and previous research [23, 68–71], results were expressed as resistant (R), intermediate (I), and sensitive (S). The zone diameter interpretive criteria are shown in Additional file 8: Table S6, with reference to CLSI [66]. In addition, multiple antimicrobial resistant isolates, showing resistance to antibiotics from three or more antimicrobial classes, were also evaluated.

### Multi-locus sequence typing and clustering of different isolates

Seven housekeeping genes (*glp*, *gmk*, *ilvD*, *pta*, *pur*, *pycA*, and *tpi*) were amplified, sequenced, and analyzed to obtain the multi-locus sequence type of the isolates according to the MLST scheme which was developed by Martin Maiden, Gus Priest, and Maggie Barker [72] on the PubMLST official website (http://pubmlst.org/bcereus/info/primers.shtml). PHYLOViZ 2.0 software (Instituto de Microbiologia, Portugal) was used to construct a minimum spanning tree of the isolates [73], to visualize the genetic diversity and inter-strain relationships. The phylogenetic relationship between different isolates was studied depending on the different STs and seven housekeeping genes, using the BioNumerics software (version 7.6; Applied Maths, Belgium) with the arithmetic mean (UPGMA) method. The strains used in the phylogenetic study contained 247 isolates identified in this study, and nine isolates (*B. cereus* ATCC14579, *Bacillus mycoides* DSM 2048, *Bacillus pseudomycoides* DSM 12442, *Bacillus weihenstephanensis* WSBC 10204, *Bacillus anthracis* ATCC4728, *Bacillus thuringiensis* ATCC10792, *Bacillus cytotoxicus* NVH 391-98 and two clinical emetic type strains *B. cereus* NC7401 and *B. cereus* F4810/72) from the *B. cereus* MLST database (www.mlstoslo.uio.no/index.html) or NCBI genome database (https://www.ncbi.nlm.nih.gov/genome/). The names of isolates, MLST sequence type (ST), MLST clonal complex (CC), antibiotic resistance patterns, and presence of toxin genes were also included.

## Supplementary information

**Additional file 1: Table S1.** Prevalence of toxin genes in *B. cereus* isolated from edible fungi in China.

**Additional file 2: Table S2.** The diameters of inhibition zones in antimicrobial susceptibility test. Among the zone diameter interpretive criteria for the antimicrobials used, the minimum value for the antimicrobial resistance interpretive criteria is 10 mm. When there is no bacteriostatic zone or the bacteriostatic zone diameter is less than 8 mm, the results are directly recorded as “R”.

**Additional file 3: Table S3.** Results of antibiotic resistance test for *B. cereus* isolates in the study.

**Additional file 4: Table S4.** The MLST typing results, and the profiles of antibiotic resistance and toxin genes of all isolates. “*” represents a new ST.

**Additional file 5: Figure S1.** Phylogenetic analysis of 247 *B. cereus* isolates from edible fungi and eight type strains based on the MLST typing results. A cut-off value of 43% similarity was applied to define the clusters. ATCC14579: *B. cereus* ATCC14579; DSM 2048: *Bacillus mycoides* DSM 2048; DSM 12442: *Bacillus pseudomycoides* DSM 12442; WSBC 10204: *Bacillus weihenstephanensis* WSBC 10204; ATCC4728: *Bacillus anthracis* ATCC4728; ATCC10792: *Bacillus thuringiensis* ATCC10792; NVH 391-98: *Bacillus cytotoxicus* NVH 391-98; NC7401: clinical emetic type strain *B. cereus* NC7401; F4810/72: clinical emetic type strain *B. cereus* F4810/72.

**Additional file 6: Figure S2.** Sampling cities where the edible fungi were collected. Sampling plan design and this sampling map were all done by ourselves.

**Additional file 7: Table S5.** Primers used in this study.

**Additional file 8: Table S6.** Zone diameter interpretive criteria for the antimicrobials used in this study.

## Data Availability

Genetic polymorphisms data used for Additional file 5: Figure S1 has been list in Additional file 4: Table S4. The sequence type and corresponding DNA sequences of seven housekeeping genes (*glp*, *gmk*, *ilvD*, *pta*, *pur*, *pycA*, and *tpi*) were all available and can be downloaded from the public MLST database (https://pubmlst.org/bcereus/) according to the STs of the isolates list in Additional file 4: Table S4. All other data generated or analyzed during this study are included in this manuscript and its supplementary information files.

## Abbreviations

MPN: Most probable number
Hbl: Hemolysin BL
Nhe: Nonhemolytic enterotoxin
CytK: Cytotoxin K
*cesB*: Cereulide synthetase gene *B*
AMP: Ampicillin
P: Penicillin
AMC: Amoxicillin-clavulanic acid
KF: Cephalothin
FOX: Cefoxitin
RD: Rifampin
IPM: Imipenem
CN: Gentamicin
CIP: Ciprofloxacin
C: Chloramphenicol
DA: Clindamycin
QD: Quinupristin-dalfopristin
FD: Nitrofurantoin
MLST: Multi-locus sequence typing
CCs: Clonal complexes
CFU: Colony-forming unit
PBS: Phosphate buffered saline
TSB: Tryptic soy broth
MYP: Mannitol egg yolk polymyxin
PCR: Polymerase chain reaction
MH: Mueller-hinton
R: Resistant
I: Intermediate
S: Sensitive
MAR: Multiple antimicrobial resistance
