# Supplementary material for: Assessment and molecular characterization of Bacillus cereus isolated from edible fungi in China
Source: BMC Microbiol. 2020 Oct 14;20:310. doi: 10.1186/s12866-020-01996-0 (PMC7557095; doi:10.1186/s12866-020-01996-0)
Supplement: Supplementary file 1 — Additional file 1: Table S1. Prevalence of toxin genes in B. cereus isolated from edible fungi in China. [file 12866_2020_1996_MOESM1_ESM.docx]

**Additional file 1: Table S1** Prevalence of toxin genes in *B. cereus* isolated from edible fungi in China.

| **Toxigenic genes** | **Number of strains (%) positive for target gene** |
| --- | --- |
| Hemolysin BL genes |  |
| *hblA* | 197 (80%) |
| *hblC* | 219 (89%) |
| *hblD* | 224 (91%) |
| *hblACD* | 191 (77%) |
| Non-hemolytic enterotoxin genes |  |
| *nheA* | 247 (100%) |
| *nheB* | 222 (90%) |
| *nheC* | 241 (98%) |
| *nheABC* | 219 (89%) |
| Cytotoxin K gene |  |
| *cytK* | 204 (83%) |
| Cereulide synthetase gene |  |
| *cesB* | 7 (3%) |
| All eight toxin genes | 4 (2%) |
