# Supplementary material for: Assessment and molecular characterization of Bacillus cereus isolated from edible fungi in China
Source: BMC Microbiol. 2020 Oct 14;20:310. doi: 10.1186/s12866-020-01996-0 (PMC7557095; doi:10.1186/s12866-020-01996-0)
Supplement: Supplementary file 3 — Additional file 3: Table S3. Results of antibiotic resistance test for B. cereus isolates in the study. [file 12866_2020_1996_MOESM3_ESM.docx]

**Additional file 3: Table S3** Results of antibiotic resistant tests for in this study

| Category | Antimicrobial Class | Antimicrobials | *Bacillus cereus* (n = 247) | | |
| --- | --- | --- | --- | --- | --- |
|  |  |  | Resistant | Intermediate | Sensitive |
| β-Lactams | Ⅰ | Penicillins |  |  |  |
|  |  | Ampicillin (10 μg) | 247 (100.0%) | 0 (0.0%) | 0 (0.0%) |
|  |  | Penicillin (10 units) | 247 (100.0%) | 0 (0.0%) | 0 (0.0%) |
|  | Ⅱ | β-Lactam / β-lactamase inhibitor combinations |  |  |  |
|  |  | Amoxicillin-clavulanic acid (20 μg/10 μg) | 245 (99.2%) | 0 (0.0%) | 2 (0.8%) |
|  | Ⅲ | Cephems (parenteral) |  |  |  |
|  |  | Cephalothin (30 μg) | 211 (85.4%) | 23 (9.3%) | 13 (5.3%) |
|  |  | Cefoxitin (30 μg) | 227 (91.9%) | 0 (0.0%) | 20 (8.1%) |
|  |  | Cefotetan (30 μg) | 47 (19.0%) | 28 (11.3%) | 172 (69.6%) |
|  | Ⅳ | Penems |  |  |  |
|  |  | Imipenem (10 μg) | 1 (0.4%) | 1 (0.4%) | 245 (99.2%) |
| Non–β-Lactams | Ⅴ | Aminoglycosides |  |  |  |
|  |  | Gentamicin (10 μg) | 3 (1.2%) | 2 (0.8%) | 242 (98.0%) |
|  |  | Kanamycin (30 μg) | 0 (0.0%) | 32 (13.0%) | 215 (87.0%) |
|  | Ⅵ | Macrolides |  |  |  |
|  |  | Erythromycin (15 μg) | 3 (1.2%) | 99 (40.1%) | 145 (58.7%) |
|  | Ⅶ | Ketolide |  |  |  |
|  |  | Telithromycin (15 μg) | 7 (2.8%) | 39 (15.8%) | 201 (81.4%) |
|  | Ⅷ | Glycopeptides |  |  |  |
|  |  | Teicoplanin (30 μg) | 8 (3.2%) | 37 (15.0%) | 202 (81.8%) |
|  | Ⅸ | Quinolones |  |  |  |
|  |  | Ciprofloxacin (5 μg) | 3 (1.2%) | 14 (5.7%) | 230 (93.1%) |
|  | Ⅹ | Phenylpropanol |  |  |  |
|  |  | Chloramphenicol (30 μg) | 2 (0.8%) | 14 (5.7%) | 231 (93.5%) |
|  | Ⅺ | Tetracyclines |  |  |  |
|  |  | Tetracycline (30 μg) | 17 (6.9%) | 49 (19.8%) | 181 (73.3%) |
|  | Ⅻ | Folate pathway inhibitors |  |  |  |
|  |  | Trimethoprim-Sulfamethoxazole (1.25 μg/23.75 μg) | 56 (22.7%) | 20 (8.1%) | 171 (69.2%) |
|  | XIII | Lincosamides |  |  |  |
|  |  | Clindamycin (2 μg) | 10 (4.0%) | 207 (83.8%) | 30 (12.1%) |
|  | XIV | Ansamycins |  |  |  |
|  |  | Rifampin (5 μg) | 225 (91.1%) | 15 (6.1%) | 7 (2.8%) |
|  | XV | Streptogramins |  |  |  |
|  |  | Quinupristin-dalfopristin (15 μg) | 20 (8.1%) | 178 (72.1%) | 49 (19.8%) |
|  | XVI | Nitrofurans |  |  |  |
|  |  | Nitrofurantoin (300 μg) | 17 (6.9%) | 87 (35.2%) | 143 (57.9%) |
| Multiple antimicrobial resistance |  | ≥ 3 Antimicrobials | 246 (99.6%) | - | - |
|  |  | ≥ 4 Antimicrobials | 227 (91.9%) | - | - |
|  |  | ≥ 5 Antimicrobials | 97 (39.3%) | - | - |
