# Supplementary material for: Assessment and molecular characterization of Bacillus cereus isolated from edible fungi in China
Source: BMC Microbiol. 2020 Oct 14;20:310. doi: 10.1186/s12866-020-01996-0 (PMC7557095; doi:10.1186/s12866-020-01996-0)
Supplement: Supplementary file 4 — Additional file 4: Table S4. The MLST typing results, and the profiles of antibiotic resistance and toxin genes of all isolates. “*” represents a new ST. [file 12866_2020_1996_MOESM4_ESM.docx]

**Additional file 4: Table S4.** The MLST typing results, and the profiles of antibiotic resistance and toxin genes of all isolates.

| **Number** | **Isolates** | **ST** | **Clonal complex** | **Antibiotic resistance pattern** | **Profile of toxin genes** |
| --- | --- | --- | --- | --- | --- |
| 1 | 21-1 | 2345 | ST-142 complex | AMP-P-AMC-KF-FOX-SXT-RD-QD | *hblA*-*hblC*-*nheA*-*nheB*-*nheC*-*cytK* |
| 2 | 21-2 | 18 | ST-18 complex | AMP-P-AMC-KF-FOX-CTT-TEC-SXT-DA-RD-QD | *hblA*-*hblC*-*hblD*-*nheA*-*nheB*-*nheC*-*cytK* |
| 3 | 21-3 | 18 | ST-18 complex | AMP-P-AMC-KF-FOX-CTT-TEC-RD | *hblA*-*hblC*-*hblD*-*nheA*-*nheB*-*nheC*-*cytK* |
| 4 | 48-1 | 2571* |  | AMP-P-AMC-KF-FOX-SXT-RD | *hblA*-*hblC*-*hblD*-*nheA*-*nheB*-*nheC*-*cytK* |
| 5 | 48-2 | 2572* |  | AMP-P-AMC-KF-FOX-SXT-QD | *hblA*-*hblC*-*hblD*-*nheA*-*nheC*-*cytK* |
| 6 | 49-1 | 26 |  | AMP-P-AMC-KF-FOX-TE-RD | *hblD*-*nheA*-*nheB*-*nheC*-*cytK*-*cesB* |
| 7 | 49-2 | 2573* | ST-142 complex | AMP-P-AMC-KF-FOX-SXT-RD | *hblA*-*hblC*-*hblD*-*nheA*-*nheB*-*nheC*-*cytK* |
| 8 | 50 | 1481 | ST-142 complex | AMP-P-AMC-KF-FOX-CTT-SXT-RD | *hblA*-*hblC*-*hblD*-*nheA*-*nheB*-*nheC*-*cytK* |
| 9 | 69-2 | 2574* |  | AMP-P-AMC-KF-FOX | *hblC*-*hblD*-*nheA*-*nheB*-*nheC*-*cytK* |
| 10 | 70-1 | 78 |  | AMP-P-AMC-KF-FOX-SXT-RD | *hblA*-*hblC*-*hblD*-*nheA*-*cytK* |
| 11 | 71 | 960 | ST-8 complex | AMP-P-AMC-KF-FOX-CTT-RD | *hblA*-*hblC*-*hblD*-*nheA*-*nheB*-*nheC*-*cytK* |
| 12 | 97 | 1483 |  | AMP-P-AMC-KF-FOX-RD | *hblA*-*hblC*-*hblD*-*nheA*-*nheB*-*nheC*-*cytK* |
| 13 | 99 | 795 | ST-205 complex | AMP-P-AMC-FOX-RD | *hblD*-*nheA*-*nheC*-*cytK* |
| 14 | 100 | 90 |  | AMP-P-AMC-FOX-TE-RD | *hblC*-*nheA*-*cytK* |
| 15 | 119 | 2281 | ST-205 complex | AMP-P-AMC-RD | *hblD*-*nheA*-*nheC* |
| 16 | 120-1 | 205 | ST-205 complex | AMP-P-AMC-KF-FOX-RD | *nheA*-*nheC*-*cytK* |
| 17 | 121-2 | 18 | ST-18 complex | AMP-P-AMC-KF-FOX-CTT-TEC-CIP-SXT-RD | *hblA*-*hblC*-*nheA*-*nheC*-*cytK* |
| 18 | 146 | 1236 | ST-142 complex | AMP-P-AMC-KF-FOX-SXT-RD | *hblA*-*hblC*-*hblD*-*nheA*-*nheB*-*nheC*-*cytK* |
| 19 | 147 | 1001 | ST-18 complex | AMP-P-AMC-KF-FOX-CTT-SXT-RD | *hblA*-*hblD*-*nheA*-*nheB*-*nheC*-*cytK* |
| 20 | 169 | 138 | ST-18 complex | AMP-P-AMC-KF-FOX-CTT-SXT-RD | *hblA*-*hblC*-*hblD*-*nheA*-*nheB*-*nheC*-*cytK* |
| 21 | 171-1 | 2150 | ST-142 complex | AMP-P-AMC-KF-FOX-CTT-RD | *hblA*-*hblC*-*hblD*-*nheA*-*nheB*-*nheC*-*cytK* |
| 22 | 171-2 | 1194 | ST-18 complex | AMP-P-AMC-KF-FOX-CTT-RD | *hblA*-*hblC*-*hblD*-*nheA*-*nheB*-*nheC*-*cytK* |
| 23 | 219 | 770 |  | AMP-P-AMC-FOX-RD-FD | *hblD*-*nheA*-*nheC*-*cytK* |
| 24 | 220-1 | 2575* | ST-97 complex | AMP-P-AMC-KF-FOX-CTT-RD | *hblA*-*hblC*-*hblD*-*nheA*-*nheB*-*nheC*-*cytK* |
| 25 | 221-1 | 2576* |  | AMP-P-AMC-KF-FOX-RD | *hblA*-*hblC*-*hblD*-*nheA*-*nheB*-*nheC* |
| 26 | 221-2 | 2577* |  | AMP-P-AMC-KF-FOX-CTT-RD | *hblA*-*hblC*-*hblD*-*nheA*-*nheB*-*nheC*-*cytK* |
| 27 | 248 | 2578* |  | AMP-P-AMC-KF-FOX-CTT-RD | *hblA*-*hblC*-*hblD*-*nheA*-*nheB*-*nheC*-*cytK* |
| 28 | 249 | 770 |  | AMP-P-AMC-RD | *hblC*-*nheA*-*nheC*-*cytK* |
| 29 | 270-3A | 205 | ST-205 complex | AMP-P-AMC-KF-FOX-RD | *nheA*-*nheC*-*cytK* |
| 30 | 285-1B | 799 | ST-142 complex | AMP-P-AMC-KF-FOX-TE-RD | *hblA*-*hblC*-*hblD*-*nheA*-*nheB*-*nheC*-*cytK* |
| 31 | Y318 | 197 | ST-23 complex | AMP-P-AMC-KF-FOX-CTT-DA-RD | *hblA*-*hblC*-*hblD*-*nheA*-*nheB*-*nheC*-*cytK* |
| 32 | 333-3A | 2579* |  | AMP-P-AMC-KF-FOX-RD | *hblA*-*hblC*-*hblD*-*nheA*-*nheB*-*nheC*-*cytK* |
| 33 | Y399 | 98 |  | AMP-P-AMC-KF-FOX-CTT-SXT-RD-QD | *hblA*-*hblC*-*hblD*-*nheA*-*nheB*-*nheC*-*cytK* |
| 34 | 470-3A | 2578* |  | AMP-P-AMC-KF-FOX-CTT-RD-QD | *hblA*-*hblC*-*hblD*-*nheA*-*nheB*-*nheC*-*cytK* |
| 35 | Y498 | 38 |  | AMP-P-AMC-KF-FOX-RD | *hblA*-*hblC*-*hblD*-*nheA*-*nheB* |
| 36 | 533-3B | 2580* | ST-142 complex | AMP-P-AMC-KF-FOX-CTT-RD | *hblA*-*hblC*-*hblD*-*nheA*-*nheB*-*nheC*-*cytK* |
| 37 | Y620 | 2581* | ST-8 complex | AMP-P-AMC-KF-FOX-CTT-RD | *hblA*-*hblC*-*hblD*-*nheA*-*nheB*-*nheC*-*cytK* |
| 38 | 633-1C | 1065 | ST-205 complex | AMP-P-AMC-KF-FOX | *nheA*-*nheC*-*cytK*-*cesB* |
| 39 | 634-1A | 611 | ST-205 complex | AMP-P-AMC-KF-FOX-RD | *nheA*-*nheC* |
| 40 | Y635 | 100 | ST-142 complex | AMP-P-AMC-KF-FOX-RD | *hblA*-*hblC*-*hblD*-*nheA*-*nheB*-*nheC*-*cytK* |
| 41 | Y650 | 1262 |  | AMP-P-AMC-KF-FOX-SXT-RD-QD-FD | *hblA*-*hblC*-*hblD*-*nheA*-*nheB*-*nheC*-*cytK* |
| 42 | 718 | 2582* | ST-18 complex | AMP-P-AMC-KF-FOX-CTT-RD | *hblA*-*hblC*-*hblD*-*nheA*-*nheB*-*nheC*-*cytK* |
| 43 | 720-2 | 395 | ST-142 complex | AMP-P-AMC-KF-FOX-RD | *hblA*-*hblC*-*hblD*-*nheA*-*nheB*-*nheC*-*cytK* |
| 44 | 770-1 | 2583* |  | AMP-P-E-TEL-TEC-C-TE-SXT-DA-RD-QD-FD | *hblA*-*hblC*-*hblD*-*nheA*-*nheB*-*nheC*-*cytK* |
| 45 | 783 | 26 |  | AMP-P-AMC-KF-FOX-RD | *hblC*-*hblD*-*nheA*-*nheB*-*nheC*-*cytK* |
| 46 | 800 | 26 |  | AMP-P-AMC-KF-FOX-RD | *hblC*-*nheA*-*nheB*-*nheC* |
| 47 | 1121 | 2584* |  | AMP-P-AMC-KF-FOX | *hblA*-*hblC*-*hblD*-*nheA*-*nheB* |
| 48 | 1171 | 1766 |  | AMP-P-AMC-KF-FOX-DA | *hblA*-*hblC*-*hblD*-*nheA*-*nheB*-*nheC* |
| 49 | 1200 | 1243 | ST-18 complex | AMP-P-AMC-KF-FOX-CTT-TE-RD | *hblA*-*hblC*-*hblD*-*nheA*-*nheB*-*nheC*-*cytK* |
| 50 | 1271-2 | 2585* |  | AMP-P-AMC-KF-FOX-RD | *hblA*-*hblC*-*hblD*-*nheA*-*nheB*-*nheC*-*cytK* |
| 51 | 1284 | 2331 | ST-205 complex | AMP-P-RD | *hblC*-*hblD*-*nheA*-*nheC*-*cytK*-*cesB* |
| 52 | 1300-1 | 4 | ST-142 complex | AMP-P-AMC-KF-FOX-RD | *hblA*-*hblC*-*hblD*-*nheA*-*nheB*-*nheC*-*cytK* |
| 53 | 1300-3 | 1723 |  | AMP-P-AMC-FOX-SXT-RD | *hblA*-*hblC*-*hblD*-*nheA*-*nheB*-*nheC*-*cytK* |
| 54 | 1369 | 2586* |  | AMP-P-AMC-KF-FOX-SXT-DA-RD | *hblA*-*hblC*-*hblD*-*nheA*-*nheC* |
| 55 | 1371-2 | 1001 | ST-18 complex | AMP-P-AMC-KF-FOX-CTT-SXT-RD-QD | *hblA*-*hblC*-*hblD*-*nheA*-*nheB*-*nheC*-*cytK* |
| 56 | 1384 | 2587* |  | AMP-P-AMC-KF-FOX-E-TEL-SXT-DA-RD-QD | *hblA*-*hblC*-*hblD*-*nheA*-*nheB*-*nheC* |
| 57 | 1385-3 | 1025 |  | AMP-P-AMC-KF-FOX-SXT-RD | *hblA*-*hblC*-*hblD*-*nheA*-*nheB*-*nheC*-*cytK* |
| 58 | 1399-1 | 1766 |  | AMP-P-AMC-KF-FOX-SXT-RD | *hblA*-*hblC*-*hblD*-*nheA*-*nheB*-*nheC*-*cytK* |
| 59 | 886 | 100 | ST-142 complex | AMP-P-AMC-KF-FOX-SXT-RD | *hblA*-*hblC*-*hblD*-*nheA*-*nheB*-*nheC* |
| 60 | Y899 | 2588* |  | AMP-P-AMC-KF-FOX-RD | *hblA*-*hblC*-*hblD*-*nheA*-*nheC* |
| 61 | 900 | 1431 | ST-142 complex | AMP-P-AMC-KF-FOX-RD | *hblA*-*hblC*-*hblD*-*nheA*-*nheB*-*nheC*-*cytK* |
| 62 | 986 | 26 |  | AMP-P-AMC-KF-FOX-RD | *nheA*-*nheB*-*nheC*-*cytK* |
| 63 | 1020 | 1001 | ST-18 complex | AMP-P-AMC-KF-FOX-CTT-SXT-RD | *hblA*-*hblC*-*hblD*-*nheA*-*nheB*-*nheC*-*cytK* |
| 64 | 1021 | 2589* | ST-142 complex | AMP-P-AMC-KF-FOX-CTT-SXT-RD | *hblA*-*hblC*-*hblD*-*nheA*-*nheB*-*nheC*-*cytK* |
| 65 | 1036-2 | 1731 |  | AMP-P-AMC-KF-FOX-SXT-RD | *hblA*-*hblC*-*hblD*-*nheA*-*nheB*-*nheC* |
| 66 | 1049 | 1708 |  | AMP-P-AMC-KF-FOX-CTT-SXT-RD | *hblA*-*hblC*-*hblD*-*nheA*-*nheB*-*nheC*-*cytK* |
| 67 | 1448 | 1595 |  | AMP-P-AMC-KF-FOX-RD | *hblA*-*hblC*-*hblD*-*nheA*-*nheB*-*nheC*-*cytK* |
| 68 | 1500-4C | 18 | ST-18 complex | AMP-P-AMC-KF-FOX-TE-RD | *hblA*-*hblC*-*hblD*-*nheA*-*nheB*-*nheC*-*cytK* |
| 69 | 1521-1B | 1764 |  | AMP-P-AMC-KF-FOX-RD | *hblA*-*hblC*-*hblD*-*nheA*-*nheB*-*nheC*-*cytK* |
| 70 | 1550-2C | 4 | ST-142 complex | AMP-P-AMC-KF-FOX-RD | *hblA*-*hblD*-*nheA*-*nheB*-*nheC*-*cytK* |
| 71 | 1569-1B | 462 | ST-205 complex | AMP-P-AMC-KF-FOX | *hblC*-*nheA*-*nheC* |
| 72 | Y1570 | 2590* |  | AMP-P-AMC-KF-FOX-RD | *hblA*-*hblC*-*hblD*-*nheA*-*nheB*-*nheC* |
| 73 | 1571-1A | 2591* |  | AMP-P-AMC-KF-FOX-CN | *hblC*-*hblD*-*nheA*-*nheC* |
| 74 | 1597-2C | 1150 |  | AMP-P-AMC-KF-FOX-SXT-RD | *hblA*-*hblC*-*hblD*-*nheA*-*nheB*-*nheC*-*cytK* |
| 75 | 1598-3A | 78 |  | AMP-P-AMC-KF-SXT-RD | *hblA*-*hblC*-*hblD*-*nheA*-*nheC*-*cytK* |
| 76 | 1619-2C | 4 | ST-142 complex | AMP-P-AMC-KF-TE-RD | *hblC*-*hblD*-*nheA*-*nheB*-*nheC*-*cytK* |
| 77 | 1619-3C | 78 |  | AMP-P-AMC-KF-FOX-RD | *hblA*-*hblC*-*hblD*-*nheA*-*nheC*-*cytK* |
| 78 | 1620-3C-1 | 4 | ST-142 complex | AMP-P-AMC-KF-FOX-RD | *hblA*-*hblC*-*hblD*-*nheA*-*nheB*-*nheC*-*cytK* |
| 79 | 1621-1C-1 | 1471 |  | AMP-P-AMC-KF-FOX-CN | *hblA*-*hblC*-*hblD*-*nheA*-*nheB*-*nheC*-*cytK* |
| 80 | 1621-1B | 1214 |  | AMP-P-AMC-KF-FOX-RD | *hblA*-*hblC*-*hblD*-*nheA*-*nheB*-*nheC*-*cytK* |
| 81 | 1647-2A | 2592* |  | AMP-P-AMC-KF-FOX-CIP | *hblC*-*hblD*-*nheA*-*nheB*-*nheC*-*cytK* |
| 82 | 1649-1B | 1237 | ST-142 complex | AMP-P-AMC-KF-FOX-RD | *hblA*-*hblC*-*hblD*-*nheA*-*nheB*-*nheC*-*cytK* |
| 83 | 1650-3A | 1290 | ST-142 complex | AMP-P-AMC-KF-FOX-SXT-RD | *hblA*-*hblC*-*hblD*-*nheA*-*nheB*-*nheC*-*cytK* |
| 84 | Y1670 | 177 |  | AMP-P-AMC-KF-FOX-RD | *hblA*-*hblC*-*hblD*-*nheA*-*nheB*-*nheC*-*cytK* |
| 85 | 1671-1B | 1216 |  | AMP-P-AMC-KF-FOX-TE-RD | *hblA*-*hblC*-*hblD*-*nheA*-*nheB*-*nheC*-*cytK* |
| 86 | 1698-1C | 1001 | ST-18 complex | AMP-P-AMC-KF-FOX-RD | *hblA*-*hblC*-*hblD*-*nheA*-*nheB*-*nheC*-*cytK* |
| 87 | 1699-2C | 2593* | ST-142 complex | AMP-P-AMC-KF-FOX-SXT-RD | *hblA*-*hblC*-*hblD*-*nheA*-*nheB*-*nheC*-*cytK* |
| 88 | 1720-2B | 770 |  | AMP-P-AMC-FOX-RD-FD | *hblA*-*nheA*-*nheB*-*nheC*-*cytK* |
| 89 | 1721-1A | 2594* |  | AMP-P-AMC-KF-FOX-SXT-RD | *hblA*-*hblC*-*hblD*-*nheA*-*nheB*-*nheC*-*cytK* |
| 90 | 1769-1C | 1814 | ST-142 complex | AMP-P-AMC-KF-FOX-RD-QD | *hblA*-*hblC*-*hblD*-*nheA*-*nheB*-*nheC*-*cytK* |
| 91 | 1769-1B | 1814 | ST-142 complex | AMP-P-AMC-KF-FOX-RD | *hblA*-*hblC*-*hblD*-*nheA*-*nheB*-*nheC*-*cytK* |
| 92 | 1770-2B | 1237 | ST-142 complex | AMP-P-AMC-KF-FOX-DA-RD | *hblA*-*hblC*-*hblD*-*nheA*-*nheB*-*nheC*-*cytK* |
| 93 | 1771-1C | 2595* |  | AMP-P-AMC-FOX-E-TEL-RD-FD | *hblA*-*hblC*-*hblD*-*nheA*-*nheB*-*nheC*-*cytK* |
| 94 | 1796-1B | 1150 |  | AMP-P-AMC-RD | *hblA*-*hblC*-*hblD*-*nheA*-*nheB*-*nheC*-*cytK* |
| 95 | Y1799 | 465 | ST-18 complex | AMP-P-AMC-KF-FOX-TE-RD | *hblA*-*hblC*-*hblD*-*nheA*-*nheB*-*nheC*-*cytK* |
| 96 | 1820-2A | 4 | ST-142 complex | AMP-P-AMC-KF-FOX-RD | *hblA*-*hblC*-*hblD*-*nheA*-*nheB*-*nheC*-*cytK* |
| 97 | 1820-3A | 465 | ST-18 complex | AMP-P-AMC-KF-FOX-TEC-RD | *hblA*-*hblC*-*hblD*-*nheA*-*nheB*-*nheC*-*cytK* |
| 98 | Y1821 | 1766 |  | AMP-P-AMC-KF-FOX-TEC-RD | *hblA*-*hblC*-*hblD*-*nheA*-*nheB*-*nheC* |
| 99 | 1846-2C | 4 | ST-142 complex | AMP-P-AMC-KF-FOX-RD | *hblA*-*hblC*-*hblD*-*nheA*-*nheB*-*nheC*-*cytK* |
| 100 | 1846-2A | 770 |  | AMP-P-AMC-FOX-RD-FD | *nheA*-*nheB*-*nheC*-*cytK* |
| 101 | 1848-2A | 2596* |  | AMP-P-AMC-KF-FOX-RD | *hblA*-*hblC*-*hblD*-*nheA*-*nheB*-*nheC*-*cytK* |
| 102 | 1950-1 | 1241 |  | AMP-P-AMC-KF-FOX-RD | *hblA*-*hblC*-*hblD*-*nheA*-*nheB*-*nheC*-*cytK* |
| 103 | 1971 | 2597* | ST-23 complex | AMP-P-AMC-KF-FOX-CTT-RD | *hblA*-*hblC*-*hblD*-*nheA*-*nheB*-*nheC*-*cytK* |
| 104 | 1996-3 | 2598* |  | AMP-P-AMC-KF-FOX-RD | *hblA*-*hblC*-*hblD*-*nheA*-*nheB*-*nheC*-*cytK*-*cesB* |
| 105 | 1998-1 | 243 | ST-97 complex | AMP-P-AMC-KF-FOX-RD | *hblA*-*hblC*-*hblD*-*nheA*-*nheB*-*nheC*-*cytK* |
| 106 | 1998-2 | 2599* |  | AMP-P-AMC-KF-FOX-CTT-RD | *hblA*-*hblC*-*hblD*-*nheA*-*nheB*-*nheC*-*cytK* |
| 107 | 2000-2 | 104 |  | AMP-P-AMC-KF-FOX-RD | *hblA*-*hblC*-*hblD*-*nheA*-*nheB*-*nheC*-*cytK* |
| 108 | 2019-1 | 2600* | ST-18 complex | AMP-P-AMC-KF-FOX-RD | *hblA*-*hblC*-*hblD*-*nheA*-*nheB*-*nheC*-*cytK* |
| 109 | 2020-1 | 104 |  | AMP-P-AMC-KF-FOX-SXT-RD-QD | *hblA*-*hblC*-*hblD*-*nheA*-*nheB*-*nheC*-*cytK* |
| 110 | 2021-2 | 770 |  | AMP-P-AMC-RD | *hblC*-*nheA*-*nheB*-*nheC*-*cytK* |
| 111 | 2035-2 | 2601* |  | AMP-P-AMC-KF-FOX-RD | *hblA*-*hblD*-*nheA*-*nheB*-*nheC*-*cytK* |
| 112 | 2035-4 | 177 |  | AMP-P-AMC-KF-FOX-RD | *hblA*-*hblC*-*hblD*-*nheA*-*nheB*-*nheC*-*cytK* |
| 113 | 2036 | 770 |  | AMP-P-AMC-FOX-TE-RD | *hblC*-*hblD*-*nheA*-*nheB*-*nheC*-*cytK* |
| 114 | 2049-1 | 1317 |  | AMP-P-AMC-KF-FOX-RD | *hblA*-*hblC*-*hblD*-*nheA*-*nheB*-*nheC*-*cytK*-*cesB* |
| 115 | 2049-2 | 1418 |  | AMP-P-AMC-KF-FOX-RD | *hblC*-*hblD*-*nheA*-*nheB*-*nheC*-*cytK* |
| 116 | 2050-1 | 770 |  | AMP-P-AMC-RD-FD | *hblC*-*hblD*-*nheA*-*nheB*-*nheC*-*cytK* |
| 117 | 2069-2 | 770 |  | AMP-P-AMC-FOX-RD | *hblC*-*hblD*-*nheA*-*nheB*-*nheC*-*cytK* |
| 118 | 2069-3 | 1418 |  | AMP-P-AMC-KF-RD-QD | *hblC*-*hblD*-*nheA*-*nheB*-*nheC* |
| 119 | 2069-4 | 177 |  | AMP-P-AMC-KF-FOX-RD | *hblA*-*hblC*-*hblD*-*nheA*-*nheB*-*nheC*-*cytK* |
| 120 | 2070 | 2602* |  | AMP-P-AMC-KF-FOX-TEL-RD | *hblA*-*hblC*-*hblD*-*nheA*-*nheB*-*nheC*-*cytK* |
| 121 | 2071-2 | 111 | ST-111 complex | AMP-P-AMC-KF-FOX-RD-QD | *hblA*-*hblC*-*hblD*-*nheA*-*nheB*-*nheC*-*cytK* |
| 122 | 2100-1 | 2603* |  | AMP-P-AMC-FOX | *hblD*-*nheA*-*nheB*-*nheC* |
| 123 | 2100-2 | 2604* |  | AMP-P-AMC-KF-FOX-RD | *hblA*-*hblC*-*hblD*-*nheA*-*nheB*-*nheC* |
| 124 | 2120-1 | 770 |  | AMP-P-AMC-FOX-TEL-RD-FD | *nheA*-*nheB*-*nheC*-*cytK* |
| 125 | 2121-1 | 770 |  | AMP-P-AMC-FOX-TEL-RD-FD | *nheA*-*nheB*-*nheC*-*cytK* |
| 126 | 2149 | 1194 | ST-18 complex | AMP-P-AMC-KF-FOX-RD | *hblA*-*hblC*-*hblD*-*nheA*-*nheB*-*nheC*-*cytK* |
| 127 | 2150-1 | 1892 |  | AMP-P-AMC-KF-FOX-RD | *hblA*-*hblC*-*hblD*-*nheA*-*nheB*-*nheC* |
| 128 | 2185 | 104 |  | AMP-P-AMC-KF-FOX-SXT-RD-QD-FD | *hblA*-*hblC*-*hblD*-*nheA*-*nheB*-*nheC*-*cytK* |
| 129 | 2219 | 395 | ST-142 complex | AMP-P-AMC-KF-FOX-RD | *hblA*-*hblC*-*hblD*-*nheA*-*nheB*-*nheC* |
| 130 | 2221 | 2259 | ST-18 complex | AMP-P-AMC-KF-FOX-RD | *hblA*-*hblC*-*hblD*-*nheA*-*nheB*-*nheC*-*cytK* |
| 131 | 2246-1 | 476 |  | AMP-P-AMC-FOX-CTT | *nheA*-*nheB*-*nheC* |
| 132 | 2246-2 | 2605* | ST-18 complex | AMP-P-AMC-KF-FOX-RD | *hblA*-*hblC*-*hblD*-*nheA*-*nheB*-*nheC*-*cytK* |
| 133 | 2271 | 2606* |  | AMP-P-AMC-KF-FOX-RD-QD-FD | *hblA*-*hblC*-*hblD*-*nheA*-*nheB*-*nheC*-*cytK* |
| 134 | 2296 | 1265 | ST-23 complex | AMP-P-AMC-KF-FOX-CTT-RD | *hblA*-*hblC*-*hblD*-*nheA*-*nheB*-*nheC* |
| 135 | 2297-1 | 1214 |  | AMP-P-AMC-KF-FOX-RD | *hblA*-*hblC*-*hblD*-*nheA*-*nheB*-*nheC*-*cytK* |
| 136 | 2297-2 | 2607* | ST-23 complex | AMP-P-AMC-KF-FOX-RD | *hblA*-*hblC*-*hblD*-*nheA*-*nheB*-*nheC*-*cytK* |
| 137 | 2299 | 770 |  | AMP-P-AMC-FOX-RD | *nheA*-*nheB*-*nheC*-*cytK* |
| 138 | 2300 | 2608* |  | AMP-P-AMC-KF-FOX | *hblD*-*nheA*-*nheB*-*nheC* |
| 139 | 2321 | 142 | ST-142 complex | AMP-P-AMC-KF-FOX-RD | *hblA*-*hblC*-*hblD*-*nheA*-*nheB*-*nheC*-*cytK* |
| 140 | 2347-1 | 465 | ST-18 complex | AMP-P-AMC-KF-FOX-TEC-RD | *hblA*-*hblC*-*hblD*-*nheA*-*nheB*-*nheC*-*cytK* |
| 141 | 2347-2 | 2609* |  | AMP-P-AMC-KF-FOX | *hblD*-*nheA*-*nheB*-*nheC* |
| 142 | 2348 | 2610* |  | AMP-P-AMC-KF-FOX-RD | *hblA*-*hblC*-*hblD*-*nheA*-*nheB*-*nheC*-*cytK* |
| 143 | 2350-1 | 2010 | ST-142 complex | AMP-P-AMC-KF-FOX-SXT-RD | *hblA*-*hblC*-*hblD*-*nheA*-*nheB*-*nheC*-*cytK* |
| 144 | 2350-2 | 1278 |  | AMP-P-AMC-KF-FOX-RD | *hblA*-*hblC*-*hblD*-*nheA*-*nheB*-*nheC*-*cytK* |
| 145 | 2371-1 | 2502 |  | AMP-P-AMC-KF-FOX-RD | *hblA*-*hblC*-*hblD*-*nheA*-*nheB*-*nheC*-*cytK* |
| 146 | 2371-2 | 18 | ST-18 complex | AMP-P-AMC-KF-FOX-TEC-TE-RD | *hblA*-*hblC*-*hblD*-*nheA*-*nheB*-*nheC*-*cytK* |
| 147 | 2396-1 | 4 | ST-142 complex | AMP-P-AMC-KF-FOX-RD | *hblA*-*hblC*-*hblD*-*nheA*-*nheB*-*nheC*-*cytK* |
| 148 | 2396-2 | 1439 |  | AMP-P-AMC-KF-FOX-SXT-RD | *hblA*-*hblC*-*hblD*-*nheA*-*nheB*-*nheC*-*cytK* |
| 149 | 2399 | 2611* |  | AMP-P-AMC-KF-FOX-RD | *hblA*-*hblC*-*hblD*-*nheA*-*nheB*-*nheC*-*cytK* |
| 150 | 2400 | 1436 |  | AMP-P-AMC-KF-FOX-RD | *hblA*-*hblC*-*hblD*-*nheA*-*nheB*-*nheC*-*cytK* |
| 151 | 2421-1 | 2594* |  | AMP-P-AMC-KF-FOX-SXT-RD | *hblA*-*hblC*-*hblD*-*nheA*-*nheB*-*nheC*-*cytK* |
| 152 | 2421-2 | 387 |  | AMP-P-AMC-KF-FOX | *hblA*-*hblC*-*hblD*-*nheA*-*nheB*-*nheC* |
| 153 | 2446 | 2612* |  | AMP-P-AMC-KF-FOX-RD | *hblD*-*nheA*-*nheB*-*nheC* |
| 154 | 2450 | 1159 |  | AMP-P-AMC-KF-FOX-RD | *hblA*-*hblC*-*hblD*-*nheA*-*nheB*-*nheC* |
| 155 | 2471-2 | 1159 |  | AMP-P-AMC-KF-FOX-RD | *hblA*-*hblC*-*hblD*-*nheA*-*nheB*-*nheC*-*cytK* |
| 156 | 2497 | 2613* |  | AMP-P-AMC-KF-FOX-RD | *hblA*-*hblC*-*hblD*-*nheA*-*nheB*-*nheC*-*cytK* |
| 157 | 2499-1 | 1329 | ST-142 complex | AMP-P-AMC-KF-FOX-SXT-RD | *hblA*-*hblC*-*hblD*-*nheA*-*nheB*-*nheC*-*cytK* |
| 158 | 2499-3 | 1329 | ST-142 complex | AMP-P-AMC-KF-FOX-SXT-RD-QD | *hblA*-*hblC*-*hblD*-*nheA*-*nheB*-*nheC*-*cytK* |
| 159 | 2499-4 | 2614* |  | AMP-P-AMC-KF-FOX-RD | *hblA*-*hblC*-*hblD*-*nheA*-*nheB*-*nheC*-*cytK* |
| 160 | 2500 | 2615* |  | AMP-P-AMC-KF-SXT-RD | *hblA*-*hblC*-*hblD*-*nheA*-*nheB*-*nheC*-*cytK* |
| 161 | 2520-1 | 1892 |  | AMP-P-AMC-KF-FOX-CTT-RD | *hblA*-*hblC*-*hblD*-*nheA*-*nheB*-*nheC*-*cytK* |
| 162 | 2521 | 233 |  | AMP-P-AMC-FOX-RD | *hblA*-*hblC*-*hblD*-*nheA*-*nheB*-*nheC*-*cytK* |
| 163 | 2548 | 2616* |  | AMP-P-AMC-KF-FOX-SXT-RD | *hblA*-*hblC*-*hblD*-*nheA*-*nheB*-*nheC*-*cytK* |
| 164 | 2549-2 | 1293 |  | AMP-P-AMC-KF-FOX-CTT-RD | *hblA*-*hblC*-*hblD*-*nheA*-*nheB*-*nheC*-*cytK* |
| 165 | 2549-3 | 2617* |  | AMP-P-AMC-TEL-RD | *hblA*-*hblC*-*hblD*-*nheA*-*nheB*-*nheC*-*cytK* |
| 166 | 2550 | 1431 | ST-142 complex | AMP-P-AMC-KF-FOX-SXT-RD | *hblA*-*hblC*-*hblD*-*nheA*-*nheB*-*nheC*-*cytK* |
| 167 | 2571 | 2618* | ST-142 complex | AMP-P-AMC-KF-FOX-SXT-RD | *hblA*-*hblC*-*hblD*-*nheA*-*nheB*-*nheC*-*cytK* |
| 168 | 2596 | 2619* |  | AMP-P-AMC-KF-FOX-CTT-RD-QD | *hblA*-*hblC*-*hblD*-*nheA*-*nheB*-*nheC*-*cytK* |
| 169 | 2597-1 | 1723 |  | AMP-P-AMC-FOX-CTT-RD | *hblA*-*hblC*-*hblD*-*nheA*-*nheB* |
| 170 | 2597-2 | 1001 | ST-18 complex | AMP-P-AMC-KF-FOX-CTT-SXT-DA-RD | *hblA*-*hblC*-*hblD*-*nheA*-*nheB*-*nheC*-*cytK* |
| 171 | 2599 | 2612* |  | AMP-P-AMC-FOX | *hblC*-*hblD*-*nheA*-*nheB*-*nheC* |
| 172 | 2620 | 2620* |  | AMP-P-AMC-KF-FOX-SXT-RD | *hblA*-*hblC*-*hblD*-*nheA*-*nheB*-*nheC*-*cytK* |
| 173 | 2647 | 2621* |  | AMP-P-AMC-KF-FOX-RD | *hblC*-*nheA*-*nheB*-*nheC*-*cytK* |
| 174 | 2648 | 2622* | ST-205 complex | AMP-P-AMC-KF-RD-FD | *hblC*-*nheA*-*nheB*-*nheC*-*cytK* |
| 175 | 2649 | 877 | ST-142 complex | AMP-P-AMC-KF-FOX-SXT-RD | *hblA*-*hblC*-*hblD*-*nheA*-*nheB*-*nheC*-*cytK* |
| 176 | 2650-2 | 229 | ST-97 complex | AMP-P-AMC-KF-FOX-CTT-IPM-SXT-RD | *hblA*-*hblC*-*hblD*-*nheA*-*nheB*-*nheC*-*cytK* |
| 177 | 2671 | 2623* |  | AMP-P-AMC-FOX-TE-SXT-RD-QD | *hblA*-*hblC*-*hblD*-*nheA*-*nheB*-*nheC*-*cytK* |
| 178 | 2720 | 1154 |  | AMP-P-AMC-KF-FOX-CTT-RD | *hblA*-*hblC*-*hblD*-*nheA*-*nheB*-*nheC*-*cytK* |
| 179 | 2721 | 1259 |  | AMP-P-AMC-KF-FOX-CTT-TE | *hblA*-*hblC*-*hblD*-*nheA*-*nheB*-*nheC*-*cytK* |
| 180 | 2749 | 799 | ST-142 complex | AMP-P-AMC-KF-FOX-RD | *hblA*-*hblC*-*hblD*-*nheA*-*nheB*-*nheC*-*cytK* |
| 181 | 2769 | 1238 | ST-18 complex | AMP-P-AMC-KF-FOX-CTT-TE-RD | *hblA*-*hblC*-*hblD*-*nheA*-*nheB*-*nheC*-*cytK* |
| 182 | 2797-1 | 1706 |  | AMP-P-AMC-KF-FOX-RD | *hblA*-*hblC*-*hblD*-*nheA*-*nheB*-*nheC*-*cytK* |
| 183 | 2797-2 | 56 | ST-23 complex | AMP-P-AMC-KF-FOX-CTT-RD | *hblA*-*hblC*-*hblD*-*nheA*-*nheB*-*nheC*-*cytK* |
| 184 | 2850-1C | 1681 |  | AMP-P-AMC-FOX | *hblA*-*hblC*-*hblD*-*nheA*-*nheB*-*nheC*-*cytK*-*cesB* |
| 185 | 2850-2B | 2624* |  | AMP-P-AMC-FOX-RD | *hblA*-*hblC*-*hblD*-*nheA*-*nheB*-*nheC*-*cytK*-*cesB* |
| 186 | 2919-1A | 177 |  | AMP-P-AMC-KF-FOX-RD | *hblA*-*hblC*-*hblD*-*nheA*-*nheB*-*nheC*-*cytK* |
| 187 | 2920-1C | 1723 |  | AMP-P-AMC-FOX | *hblA*-*hblC*-*hblD*-*nheA*-*nheB*-*nheC* |
| 188 | 2950-1A | 2625* | ST-23 complex | AMP-P-AMC-KF-FOX | *hblA*-*hblC*-*hblD*-*nheA*-*nheB*-*nheC*-*cytK* |
| 189 | 2950-1C | 2626* | ST-142 complex | AMP-P-AMC-KF-FOX-RD | *hblA*-*hblC*-*hblD*-*nheA*-*nheB*-*nheC*-*cytK* |
| 190 | 2999 | 1607 |  | AMP-P-AMC-KF-FOX-RD | *hblA*-*hblC*-*hblD*-*nheA*-*nheC*-*cytK* |
| 191 | 2969-1A | 265 | ST-23 complex | AMP-P-AMC-KF-FOX-CTT-DA-RD | *hblA*-*hblC*-*hblD*-*nheA*-*nheB*-*nheC*-*cytK* |
| 192 | 2999-1A | 1607 |  | AMP-P-AMC-KF-FOX-RD | *hblA*-*hblC*-*hblD*-*nheA* |
| 193 | 3000-1A | 760 | ST-97 complex | AMP-P-AMC-KF-FOX-CTT-RD | *hblA*-*hblC*-*hblD*-*nheA*-*nheB*-*nheC*-*cytK* |
| 194 | 3049-1C | 205 | ST-205 complex | AMP-P-AMC-KF-FOX-RD | *hblC*-*hblD*-*nheA*-*nheB*-*nheC* |
| 195 | 3049-2B | 205 | ST-205 complex | AMP-P-AMC-FOX-RD | *hblC*-*hblD*-*nheA*-*nheB*-*nheC* |
| 196 | 3049-3C | 2627* |  | AMP-P-AMC-KF-RD | *hblA*-*hblC*-*hblD*-*nheA*-*nheB*-*nheC*-*cytK* |
| 197 | 3071-1A | 205 | ST-205 complex | AMP-P-AMC-KF-FOX-RD | *nheA*-*nheB*-*nheC* |
| 198 | 3071-2A | 205 | ST-205 complex | AMP-P-AMC-KF-FOX | *nheA*-*nheB*-*nheC*-*cytK* |
| 199 | 3098-2B | 2628* |  | AMP-P-AMC-KF-CIP-RD | *hblA*-*hblC*-*hblD*-*nheA*-*nheB*-*nheC*-*cytK* |
| 200 | 3099-1C | 2629* |  | AMP-P-AMC-KF-RD | *hblA*-*hblC*-*hblD*-*nheA*-*nheB*-*nheC*-*cytK* |
| 201 | 3099-2A | 2630* |  | AMP-P-AMC-KF-FOX-RD | *hblA*-*hblC*-*hblD*-*nheA*-*nheB*-*nheC*-*cytK* |
| 202 | 3099-2C | 1688 |  | AMP-P-AMC-KF-FOX-SXT-RD | *hblA*-*hblC*-*hblD*-*nheA*-*nheB*-*nheC*-*cytK* |
| 203 | 3197-1A | 1331 | ST-18 complex | AMP-P-AMC-KF-FOX-CTT-RD | *hblA*-*hblC*-*hblD*-*nheA*-*nheB*-*nheC*-*cytK* |
| 204 | 3198-1B | 1310 |  | AMP-P-AMC-KF-FOX-RD | *hblA*-*hblC*-*hblD*-*nheA*-*nheB*-*nheC*-*cytK* |
| 205 | 3198-1C | 2631* |  | AMP-P-AMC-KF-FOX-RD | *hblA*-*hblC*-*hblD*-*nheA*-*nheB*-*nheC*-*cytK* |
| 206 | 3199-1B | 2632* |  | AMP-P-AMC-KF-FOX-CTT-RD | *hblA*-*hblC*-*hblD*-*nheA*-*nheB*-*nheC*-*cytK* |
| 207 | 3221-1B | 2633* |  | AMP-P-AMC-KF-FOX-TE-RD | *hblA*-*hblC*-*hblD*-*nheA*-*nheB*-*nheC*-*cytK* |
| 208 | 3246-1B | 462 | ST-205 complex | AMP-P-AMC-KF-FOX-C-RD-FD | *nheA*-*nheB*-*nheC*-*cytK* |
| 209 | 3270-1A | 2634* |  | AMP-P-AMC-KF-FOX-RD-QD | *hblA*-*hblC*-*hblD*-*nheA*-*nheB*-*nheC* |
| 210 | 3321 | 1311 |  | AMP-P-AMC-KF-FOX-SXT-DA-RD-QD-FD | *hblA*-*hblC*-*hblD*-*nheA*-*nheC*-*cytK* |
| 211 | 3448 | 1690 | ST-142 complex | AMP-P-AMC-KF-FOX-RD | *hblA*-*hblC*-*hblD*-*nheA*-*nheC*-*cytK* |
| 212 | 3450-1A | 2511 |  | AMP-P-AMC-KF-FOX-RD | *hblA*-*hblC*-*hblD*-*nheA*-*nheB*-*nheC*-*cytK* |
| 213 | 3450-2A | 4 | ST-142 complex | AMP-P-AMC-KF-FOX-RD | *hblA*-*hblC*-*hblD*-*nheA*-*nheB*-*nheC*-*cytK* |
| 214 | 3450-3A | 230 | ST-23 complex | AMP-P-AMC-KF-FOX-RD | *hblA*-*hblC*-*hblD*-*nheA*-*nheB*-*nheC*-*cytK* |
| 215 | 3550-3A | 18 | ST-18 complex | AMP-P-AMC-KF-FOX-RD | *hblA*-*hblC*-*hblD*-*nheA*-*nheB*-*nheC*-*cytK* |
| 216 | 3570 | 1159 |  | AMP-P-AMC-KF-RD | *hblA*-*hblC*-*hblD*-*nheA*-*nheB*-*nheC* |
| 217 | 3570-2C | 2635* |  | AMP-P-AMC-KF-FOX-RD | *hblA*-*hblC*-*hblD*-*nheA*-*nheB*-*nheC* |
| 218 | 3598 | 2636* |  | AMP-P-AMC-KF-FOX-TE-RD | *hblA*-*hblC*-*hblD*-*nheA*-*nheB*-*nheC*-*cytK* |
| 219 | 3599 | 1210 |  | AMP-P-AMC-KF-FOX-TE-RD | *hblA*-*hblC*-*hblD*-*nheA*-*nheB*-*nheC*-*cytK* |
| 220 | 3600-1A | 387 |  | AMP-P-AMC-KF-FOX | *hblA*-*hblC*-*hblD*-*nheA*-*nheB*-*nheC* |
| 221 | 3650 | 78 |  | AMP-P-AMC-KF-FOX-RD | *hblA*-*hblC*-*hblD*-*nheA*-*nheC*-*cytK* |
| 222 | 3699-2C | 2637* | ST-18 complex | AMP-P-AMC-KF-FOX-CTT-RD | *hblA*-*hblC*-*hblD*-*nheA*-*nheB*-*nheC*-*cytK* |
| 223 | 3700-1A | 2638* |  | AMP-P-AMC-KF-FOX-RD | *hblA*-*hblC*-*hblD*-*nheA*-*nheB*-*nheC* |
| 224 | 3720-1C | 1236 | ST-142 complex | AMP-P-AMC-KF-FOX-SXT-RD | *hblA*-*hblC*-*hblD*-*nheA*-*nheB*-*nheC*-*cytK* |
| 225 | 3770-1A | 2639* |  | AMP-P-AMC-KF-FOX-SXT-RD | *hblC*-*hblD*-*nheA*-*nheB*-*nheC*-*cytK* |
| 226 | 3796 | 1311 |  | AMP-P-AMC-KF-SXT-RD | *hblA*-*hblC*-*hblD*-*nheA*-*nheB*-*nheC*-*cytK* |
| 227 | 3796-1B | 2640* |  | AMP-P-AMC-KF-FOX-SXT-RD | *hblA*-*hblC*-*hblD*-*nheA*-*nheB*-*nheC*-*cytK* |
| 228 | 3799 | 2197 |  | AMP-P-AMC-KF-FOX-RD | *hblA*-*hblC*-*hblD*-*nheA*-*nheB*-*nheC*-*cytK* |
| 229 | 3847 | 1805 | ST-97 complex | AMP-P-AMC-KF-FOX-CTT-RD | *hblA*-*hblC*-*hblD*-*nheA*-*nheB*-*nheC*-*cytK* |
| 230 | 3899 | 2641* | ST-142 complex | AMP-P-AMC-KF-RD | *hblA*-*hblC*-*hblD*-*nheA*-*nheB*-*nheC*-*cytK* |
| 231 | 3899-2A | 2642* | ST-111 complex | AMP-P-AMC-KF-FOX-CTT-RD | *hblA*-*hblC*-*hblD*-*nheA*-*nheB*-*nheC*-*cytK* |
| 232 | 3899-2B | 2643* | ST-205 complex | AMP-P-AMC-FOX-RD-FD | *hblC*-*hblD*-*nheA*-*nheB*-*nheC*-*cytK* |
| 233 | 3900-1B | 1743 |  | AMP-P-AMC-KF-FOX-RD | *hblA*-*hblC*-*hblD*-*nheA*-*nheB*-*nheC*-*cytK* |
| 234 | 3947 | 1766 |  | AMP-P-AMC-FOX-CN | *hblA*-*hblC*-*hblD*-*nheA*-*nheB*-*nheC*-*cytK* |
| 235 | 3947-1A | 2644* | ST-23 complex | AMP-P-AMC-KF-FOX-CTT-RD | *hblA*-*hblC*-*hblD*-*nheA*-*nheB*-*nheC*-*cytK* |
| 236 | 3970-1A | 2645* | ST-205 complex | AMP-P-AMC-FOX-RD | *hblA*-*hblC*-*hblD*-*nheA*-*nheB*-*nheC*-*cytK* |
| 237 | 3997-1A | 205 | ST-205 complex | AMP-P-AMC-FOX-RD-FD | *hblC*-*hblD*-*nheA*-*nheB*-*nheC*-*cytK* |
| 238 | 3997-2A | 47 |  | AMP-P-AMC-FOX-RD | *hblA*-*hblC*-*hblD*-*nheA*-*nheB*-*nheC*-*cytK* |
| 239 | 4047-1A | 2622* | ST-205 complex | AMP-P-AMC-KF-RD-FD | *hblC*-*hblD*-*nheA*-*nheB*-*nheC*-*cytK* |
| 240 | 4049-1B | 76 |  | AMP-P-AMC-KF-FOX-SXT-RD | *hblC*-*hblD*-*nheA*-*nheB*-*nheC*-*cytK* |
| 241 | 4097-1B | 2646* | ST-23 complex | AMP-P-AMC-KF-FOX-CTT-RD | *hblA*-*hblC*-*hblD*-*nheA*-*nheB*-*nheC* |
| 242 | 4148-1A | 2647* |  | AMP-P-AMC-KF-FOX-SXT-RD | *hblA*-*hblC*-*hblD*-*nheA*-*nheB*-*nheC*-*cytK* |
| 243 | 4148-1B | 960 | ST-8 complex | AMP-P-AMC-KF-FOX-CTT-RD | *hblA*-*hblC*-*hblD*-*nheA*-*nheB*-*nheC*-*cytK* |
| 244 | 4149-1A | 18 | ST-18 complex | AMP-P-AMC-KF-FOX-RD | *hblA*-*hblC*-*hblD*-*nheA*-*nheB*-*nheC*-*cytK* |
| 245 | 4197-2A | 2648* |  | AMP-P-AMC-KF-FOX-SXT-RD | *hblD*-*nheA*-*nheB*-*nheC* |
| 246 | 4197-3A | 2648* |  | AMP-P-AMC-KF-FOX-SXT-RD | *hblD*-*nheA*-*nheB*-*nheC* |
| 247 | 4200-1A | 1066 | ST-205 complex | AMP-P-AMC-FOX-RD | *hblD*-*nheA*-*nheC*-*cytK* |

“*” represents the new ST.
