# Supplementary figures and images for: Assessment and molecular characterization of Bacillus cereus isolated from edible fungi in China
Source: BMC Microbiol. 2020 Oct 14;20:310. doi: 10.1186/s12866-020-01996-0 (PMC7557095; doi:10.1186/s12866-020-01996-0)

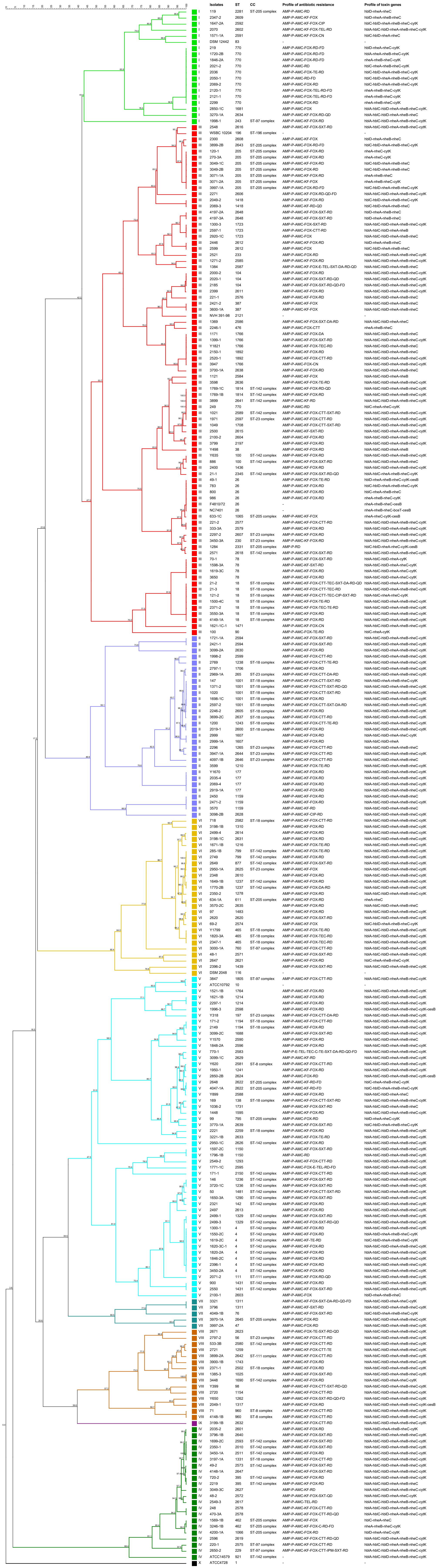

Supplement: Supplementary file 5 — Additional file 5: Figure S1. Phylogenetic analysis of 247 B. cereus isolates from edible fungi and eight type strains based on the MLST typing results. A cut-off value of 43% similarity was applied to define the clusters. ATCC14579: B. cereus ATCC14579; DSM 2048: Bacillus mycoides DSM 2048; DSM 12442: Bacillus pseudomycoides DSM 12442; WSBC 10204: Bacillus weihenstephanensis WSBC 10204; ATCC4728: Bacillus anthracis ATCC4728; ATCC10792: Bacillus thuringiensis ATCC10792; NVH 391-98: Bacillus cytotoxicus NVH 391-98; NC7401: clinical emetic type strain B. cereus NC7401; F4810/72: clinical emetic type strain B. cereus F4810/72. [file 12866_2020_1996_MOESM5_ESM.pdf]

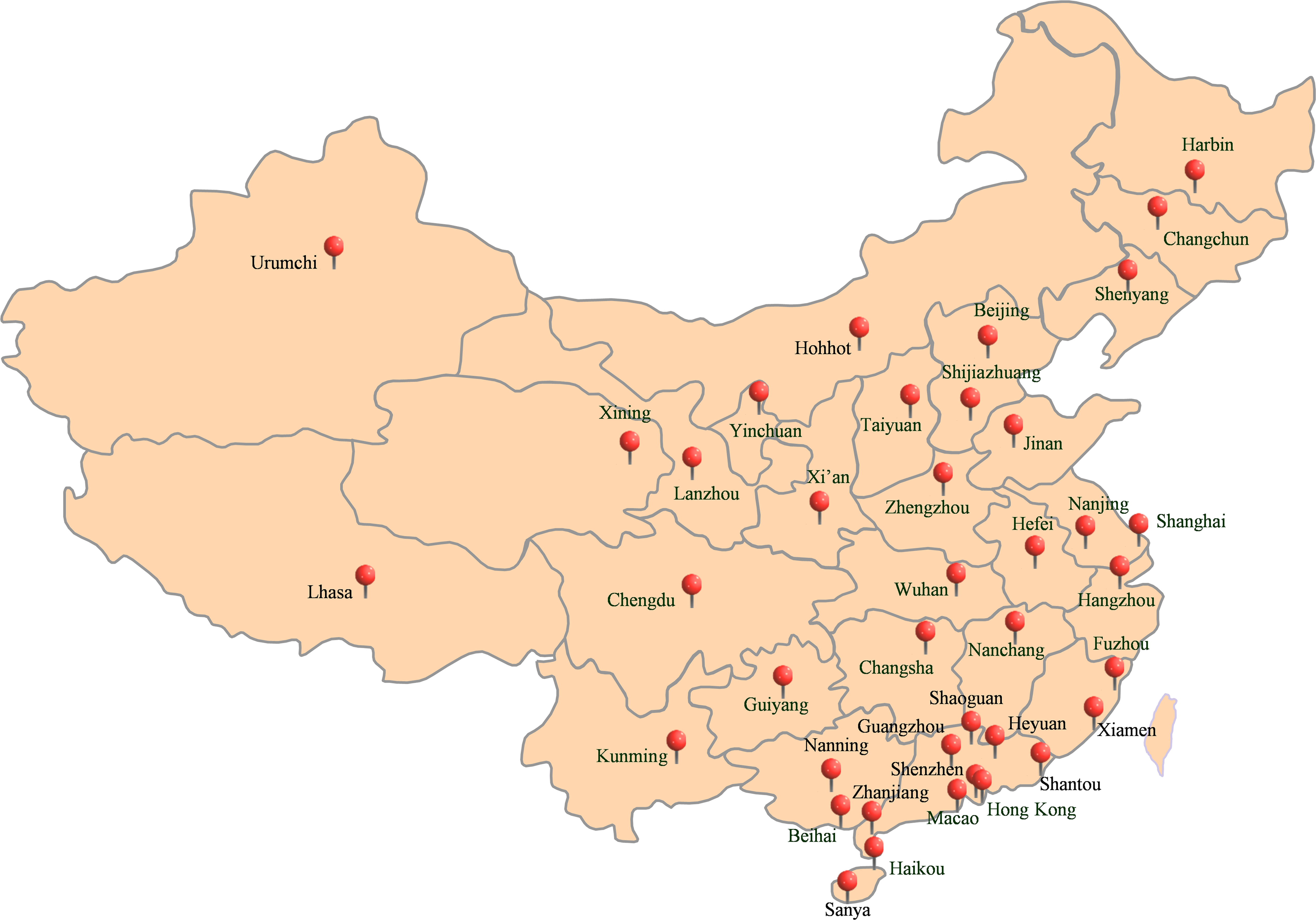

Supplement: Supplementary file 6 — Additional file 6: Figure S2. Sampling cities where the edible fungi were collected. Sampling plan design and this sampling map were all done by ourselves. [file 12866_2020_1996_MOESM6_ESM.tif]
