# Supplementary material for: Assessment and molecular characterization of Bacillus cereus isolated from edible fungi in China
Source: BMC Microbiol. 2020 Oct 14;20:310. doi: 10.1186/s12866-020-01996-0 (PMC7557095; doi:10.1186/s12866-020-01996-0)
Supplement: Supplementary file 7 — Additional file 7: Table S5. Primers used in this study. [file 12866_2020_1996_MOESM7_ESM.docx]

**Additional file 7: Table S5** Primers used in this study.

| **Primer** | **Sequence (5’-3’)** | **Target fragment length (bp)** | **Temperature used for the annealing step in PCR (℃)** | **Reference** |
| --- | --- | --- | --- | --- |
| HblA-F | GTGCAGATGTTGATGCCGAT | 320 | 55 | ([Hansen and Hendriksen, 2001](#_ENREF_25)) |
| HblA-R | ATGCCACTGCGTGGACATAT |  |  |  |
| HblC-F | AATGGTCATCGGAACTCTAT | 750 | 55 | ([Hansen and Hendriksen, 2001](#_ENREF_25)) |
| HblC-R | CTCGCTGTTCTGCTGTTAAT |  |  |  |
| HblD-F | AATCAAGAGCTGTCACGAAT | 430 | 55 | ([Hansen and Hendriksen, 2001](#_ENREF_25)) |
| HblD-R | CACCAATTGACCATGCTAAT |  |  |  |
| NheA-F | TACGCTAAGGAGGGGCA | 500 | 55 | ([Hansen and Hendriksen, 2001](#_ENREF_25)) |
| NheA-R | GTTTTTATTGCTTCATCGGCT |  |  |  |
| NheB-F | CTATCAGCACTTATGGCAG | 770 | 55 | ([Hansen and Hendriksen, 2001](#_ENREF_25)) |
| NheB-R | ACTCCTAGCGGTGTTCC |  |  |  |
| NheC-F | CGGTAGTGATTGCTGGG | 583 | 55 | ([Hansen and Hendriksen, 2001](#_ENREF_25)) |
| NheC-R | CAGCATTCGTACTTGCCAA |  |  |  |
| cytK-F | AAAATGTTTAGCATTATCCGCTGT | 238 | 55 | (Oltuszak-Walczak and Walczak, 2013) |
| cytK-R | ACCAGTTGTATTAATAACGGCAATC |  |  |  |
| cesB-F | GGTGACACATTATCATATAAGGTG | 1271 | 58 | (Ehling-Schulz et al., 2005) |
| cesB-R | GTAAGCGAACCTGTCTGTAACAACA |  |  |  |
| glpF-F | GCGTTTGTGCTGGTGTAAGT | 549 | 59 | PubMLST (http://pubmlst.org/bcereus/info/primers.shtml) |
| glpF-R | CTG CAATCGGAAGGAAGAAG |  |  |  |
| gmk-F | TTAAGTGAGGAAGGGTAGG | 600 | 56 |  |
| gmk-R | AATGTTCACCAACCACAA |  |  |  |
| ilvD-F | GGGCAAACATTAAGAGAA | 556 | 58 |  |
| ilvD-R | TTCTGGTCGTTTCCATTC |  |  |  |
| pta-F | AGAGCGTTTAGCAAAAGAA | 576 | 56 |  |
| pta-R | CAATGCGAGTTGCTTCTA |  |  |  |
| pur-F | GCTGCGAAAAATCACAAA | 536 | 56 |  |
| pur-R | CACGATTCGCTGCAATAA |  |  |  |
| pycA-F | GTTAGGTGGAAACGAAAG | 550 | 57 |  |
| pycA-R | CGTCCAAGTTTATGGAAT |  |  |  |
| tpi-F | CCAGTAGCACTTAGCGAC | 553 | 58 |  |
| tpi-R | GAAACCGTCAAGAATGAT |  |  |  |
