# Supplementary material for: Assessment and molecular characterization of Bacillus cereus isolated from edible fungi in China
Source: BMC Microbiol. 2020 Oct 14;20:310. doi: 10.1186/s12866-020-01996-0 (PMC7557095; doi:10.1186/s12866-020-01996-0)
Supplement: Supplementary file 8 — Additional file 8: Table S6. Zone diameter interpretive criteria for the antimicrobials used in this study. [file 12866_2020_1996_MOESM8_ESM.docx]

**Additional file 8: Table S6.** Zone diameter interpretive criteria for the antimicrobials used in this study.

| Category | Antimicrobial Class | Antimicrobial agents | | Zone Diameter Criteria (mm) | | |
| --- | --- | --- | --- | --- | --- | --- |
|  |  |  |  | Resistant | Intermediate | Sensitive |
| β-Lactams | Penicillins | I | Ampicillin (10 μg) | ≤28 | — | ≥ 29 |
|  |  | Ⅱ | Penicillin (10 units) | ≤28 | — | ≥ 29 |
|  | β-Lactam/β-lactamase inhibitor combinations | Ⅲ | Amoxicillin-clavulanic acid (20 μg/10μg) | ≤19 | — | ≥ 20 |
|  | Cephems (parenteral) | IV | Cephalothin (30 μg) | ≤14 | 15-17 | ≥ 18 |
|  |  | V | Cefoxitin (30 μg) | ≤21 | — | ≥ 22 |
|  |  | VI | Cefotetan (30 μg) | ≤12 | 13-15 | ≥ 16 |
|  | Penems | VII | Imipenem (10 μg) | ≤13 | 14-15 | ≥ 16 |
| Non β-Lactams | Aminoglycosides | VIII | Gentamicin (10 μg) | ≤12 | 13-14 | ≥ 15 |
|  |  | IX | Kanamycin (30 μg) | ≤13 | 14-17 | ≥ 18 |
|  | Macrolides | X | Erythromycin (15 μg) | ≤13 | 14-22 | ≥ 23 |
|  | Ketolide | XI | Telithromycin (15 μg) | ≤18 | 19-21 | ≥ 22 |
|  | Glycopeptides | XII | Teicoplanin (30 μg) | ≤10 | 11-13 | ≥ 14 |
|  | Quinolones | XIII | Ciprofloxacin (5 μg) | ≤15 | 16-20 | ≥ 21 |
|  | Phenylpropanol | XIV | Chloramphenicol (30 μg) | ≤12 | 13-17 | ≥ 18 |
|  | Tetracyclines | XV | Tetracycline (30 μg) | ≤14 | 15-18 | ≥ 19 |
|  | Folate pathway inhibitors | XVI | Trimethoprim-Sulfamethoxazole (1.25μg/23.75 μg) | ≤10 | 11-15 | ≥ 16 |
|  | Lincosamides | XVII | Clindamycin (2 μg) | ≤14 | 15-20 | ≥ 21 |
|  | Ansamycins | XVIII | Rifampin (5 μg) | ≤16 | 17-19 | ≥ 20 |
|  | Streptogramins | XIX | Quinupristin-dalfopristin  (15 μg) | ≤15 | 16-18 | ≥ 19 |
|  | Nitrofurans | XX | Nitrofurantoin (300 μg) | ≤14 | 15-16 | ≥ 17 |
